# Covalent inhibitors of the PI3Kα RAS binding domain impair tumor growth driven by RAS and HER2

**DOI:** 10.1126/science.adv2684

**Published:** 2025-10-09

**Authors:** Joseph E. Klebba, Nilotpal Roy, Steffen M. Bernard, Stephanie Grabow, Melissa A. Hoffman, Hui Miao, Junko Tamiya, Jinwei Wang, Cynthia Berry, Antonio Esparza-Oros, Richard Lin, Yongsheng Liu, Marie Pariollaud, Holly Parker, Igor Mochalkin, Sareena Rana, Aaron N. Snead, Eric J. Walton, Taylor E. Wyrick, Erick Aitichson, Karl Bedke, Jacyln C. Brannon, Joel M. Chick, Kenneth Hee, Benjamin D. Horning, Mohamed Ismail, Kelsey N. Lamb, Wei Lin, Justine Lu, Martha K. Pastuszka, Jonathan Pollock, John J. Sigler, Mona Tomaschko, Eileen Tran, Chanyu Yue, Todd M. Kinsella, Miriam Molina-Arcas, Brian N. Cook, Gabriel M. Simon, David S. Weinstein, Julian Downward, Matthew P. Patricelli

**Affiliations:** 1 Vividion Therapeutics, 5820 Nancy Ridge Drive, San Diego, California, 92121; 2 Francis Crick Institute, 1 Midland Road, London NW1 1AT, UK

## Abstract

Genetic disruption of the RAS binding domain (RBD) of Phosphoinositide 3-kinase alpha(PI3Kα) impairs the growth of tumors driven by the small guanosine triphosphatase RAS in mice and does not impact PI3Kα’s role in insulin mediated control of glucose homeostasis. Selectively blocking the RAS-PI3Kα interaction may represent a strategy for treating RAS-dependent cancers as it would avoid the toxicity associated with inhibitors of PI3Kα lipid kinase activity. We developed compounds that bind covalently to cysteine 242 in the RBD of PI3K p110α and block RAS activation of PI3Kα activity. In mice, inhibitors slow the growth of RAS mutant tumors and Human Epidermal Growth Factor Receptor 2 (HER2) over-expressing tumors, particularly when combined with other inhibitors of the RAS/Mitogen-activated protein kinase pathway, without causing hyperglycemia.

## Introduction

Oncogenic mutations in the small guanosine triphosphatase RAS occur in 20% of human cancers, with RAS proteins activating both the mitogen-activated protein kinase (MAPK) and Phosphoinositide 3-kinase (PI3K) pathways (1–3). As each of these pathways has oncogenic potential, simultaneous activation, as occurs in mutant RAS driven cancers, generates aggressive disease. In RAS-driven cell and animal models, inhibition of both the MAPK and PI3K pathways is more efficacious than targeting the individual pathways (4); however, dose limiting toxicities in humans prevent clinical success of this strategy. Although physiological activation of the MAPK pathway is RAS dependent, the interaction between RAS and the catalytic subunit of PI3Kα, p110α, serves as an amplifier but not a primary activator of this pathway, and is less important in normal cellular regulation than it is in cancer (5).

Genetic disruption of the interaction of RAS with p110α blocks growth of mutant RAS induced lung and skin tumors in mice (6, 7). Although the interaction of RAS with p110α is disrupted in all tissues of such mice, there was no impact on fitness of adult animals, indicating that in healthy cells, RAS dependent amplification of PI3Kα signaling is expendable and RAS-independent activation of PI3K by upstream signaling factors is sufficient to maintain physiological homeostasis. Unfortunately, targeting the PI3Kα pathway with inhibitors of the catalytic activity of p110α has toxic effects that limit its therapeutic application, most commonly hyperglycemia and rash, caused by inhibition of both RAS-dependent and -independent PI3K signaling (8, 9). Regulation of glucose homeostasis by insulin is severely disrupted by p110α inhibitors, including alpelisib, which is approved for the treatment of breast cancer (10).

We developed small molecules that covalently ligate Cys^242^, adjacent to the RAS binding interface of p110α, blocking its interaction with RAS proteins. In mice, these compounds inhibited growth of RAS mutant tumors, particularly when combined with Mitogen-activated protein kinase kinase (MEK) or Kirsten rat sarcoma virus (KRAS) inhibitors. These compounds also inhibited activation of PI3Kα signalling in cells over-expressing Human Epidermal Growth Factor Receptor 2 (HER2) that appears to be independent of RAS, thus it appears that the RAS binding domain (RBD) may have a non-canonical role in this setting. The molecules were well tolerated, did not impact normal blood glucose regulation and thus may be promising candidates for cancer therapy. A related molecule has entered Phase I clinical trials in humans (NCT06804824).

## Results

### Identification of compounds that bind covalently to the RAS binding domain of p110α

**Fig. 1:**
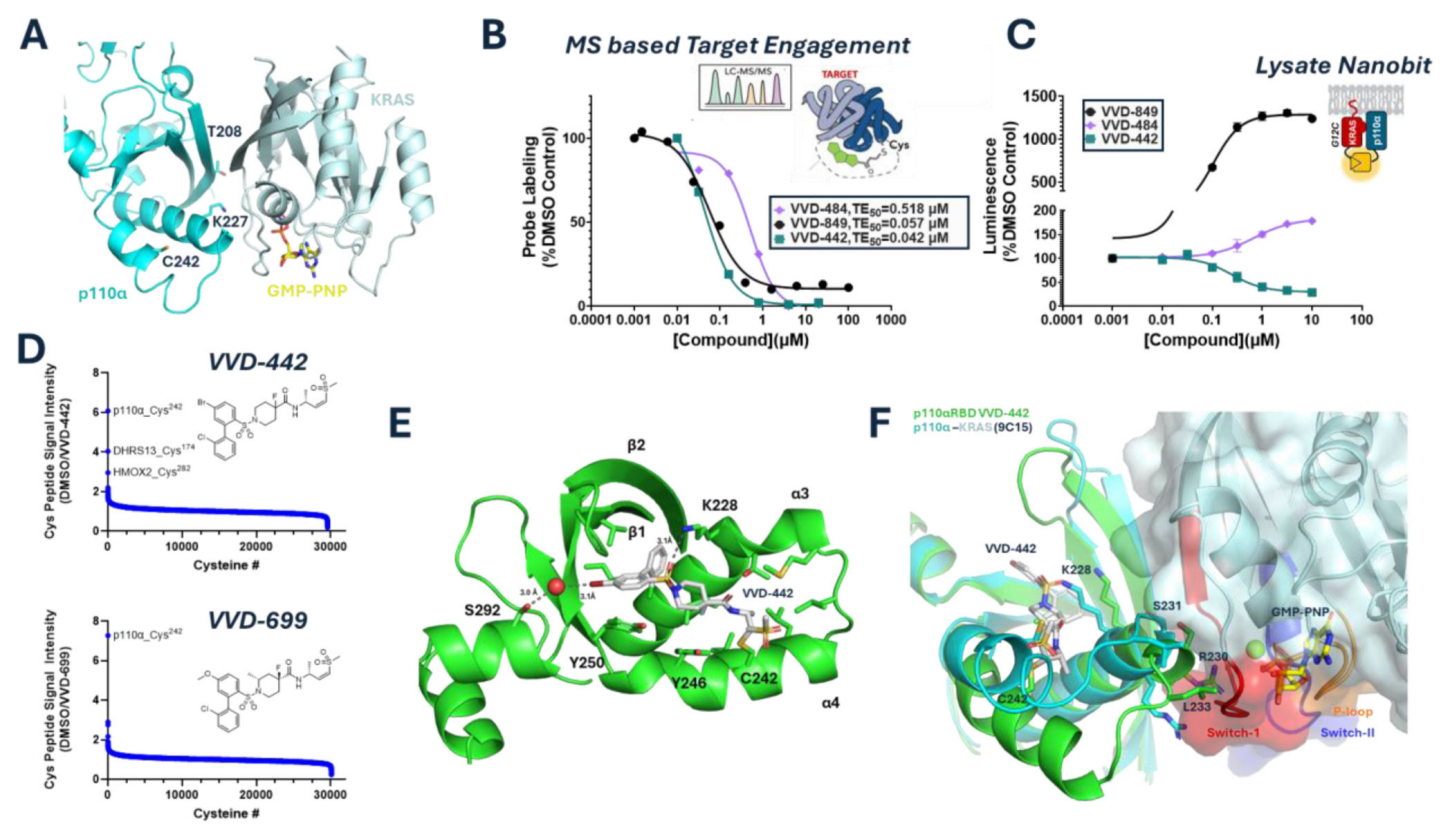
Identification of compounds that bind covalently to the RAS binding domain of p110α (A) Ribbon representation of the p110α/KRAS/GMPPNP/D927 glue complex crystal structure (PDB code 9C15). Residues Thr^208^ and Lys^227^ of p110α are located at the interface of the RBD region and KRAS and facilitate interactions between the proteins. Residue C242 is located in the α4-helix in the RBD but not on the RAS binding interface. KRAS and p110α are shown in light blue and cyan, respectively. GMPPNP is represented in yellow, and the D927 molecular glue is omitted for clarity. (B) LC-MS/MS based target engagement (TE) was calculated by comparing the peak area of probe-labeled Cys^242^ of p110α of compound treated to DMSO treated Jurkat cell lysates. (C) VVD-442, VVD-849, VVD-484 were evaluated for their ability to impact the interaction between p110α and KRAS^Gly12Cys^ using the NanoBiT protein-protein interaction assay in HEK293T cell lysates treated with compound for 1 hour (n=2 biological replicates, SD). VVD-484 was screened in this assay 3 times while VVD-849 was screened 2 times, representative data is presented. (D) Global proteomics selectivity of VVD-442 and VVD-699. Compound selectivity relative to ~30,000 cysteine containing peptides were measured at ~25-fold over TE_50_s (2 µM, 2 h live cell treatment) using TMT quantification. (E) Crystal structure of the VVD-442-RBD complex. The compound rests in a hydrophobic cleft formed by α3 and α4 and β-strands 1 and 2. (F) Superposition of the KRAS/p110α/D927 glue (9C15) and RBD/VVD-442 crystal structures showing steric clashes between residues R230, S231 and L233 of p110α in the VVD-442 complex and the switch I region of KRAS. KRAS is shown as a light blue surface with the switch I, switch II and P-loop highlighted in red, blue and orange, respectively. p110α is shown in cyan and the RBD/VVD-442 complex is shown in green with VVD-442 shown in grey sticks. GMPPNP is represented in yellow sticks and the D927 molecular glue is omitted for clarity.

Mutational studies have identified key residues, Thr^208^ and Lys^227^, within the RBD region of p110α, that facilitate interaction with RAS upon receptor tyrosine kinase-driven recruitment of PI3Kα to the plasma membrane (11, 12) (Fig. 1A). By mass-spectrometry-based chemoproteomics (13) we detected 21 cysteines on p110α, including Cys^242^, which is exclusive to p110α, in the RBD and proximal to Thr^208^ and Lys^227^ (Fig. 1A). This led us to search for covalent ligands of Cys^242^ of p110α as potential blockers of the interaction between these two proteins.

Towards this end, we used targeted chemoproteomics screening to identify compounds representing multiple chemotypes that selectively formed covalent bonds with Cys^242^ (Fig 1B. Fig. S1A). Optimization through medicinal chemistry led to the generation of potent and selective Cys^242^ engagers with diverse structural features (Fig. S1A). These compounds showed half-maximal target engagement concentrations (TE_50_), reflective of binding to p110α-Cys^242^, in the nanomolar (nM) range as measured through chemoproteomics in cell lysates treated for 1 hour. To understand the consequence of Cys^242^ binding, we developed a NanoBiT assay to measure the interaction between KRAS^Gly12Cys^ and p110α in HEK293T cell lysates (14). Briefly, this assay utilizes two inactive fragments of a luciferase enzyme, Large Bit (LgBit) and Small Bit (SmBit), which can interact when brought into proximity and regenerate a functional luciferase enzyme. When SmBit-KRAS^Gly12Cys^ and LgBit-p110α form a complex, a luminescence signal is generated and can be used to quantitatively assess the interaction between KRAS^Gly12Cys^ and p110α. This assay revealed that compounds binding to p110α-Cys^242^ fell into three different classes: blockers of the interaction, promoters of the interaction (glues), or compounds lacking effect on the interaction (silent ligands) (Fig 1C, Fig. S1A, Table S1). As our goal was to develop a small molecule that would disrupt the RAS-p110α interaction, we focused in this study on a number of blockers: VVD-442(Fig. 1C), VVD-699, VVD-844 and VVD-579 (Fig. S1A-B, Fig. S2A, Table S1). Global proteomic profiling of interaction of VVD-442 and VVD-699 against ~30,000 cellular Cys residues demonstrated that Cys^242^ of p110α was the most potently engaged target of both compounds (Fig. 1D).

To understand how our covalent ligands for Cys^242^ blocked the interaction between RAS and p110α, we determined the X-ray co-crystal structure of VVD-442 covalently bound to the RBD of p110α at 2.83 Å resolution (Fig 1E, Fig. S1C and Table S2). The compound covalently bound to Cys^242^ in a hydrophobic groove between α-helices 3 and 4. The central sulfonamide formed a hydrogen bond with the terminal amide of Lys^228,^ and the bromine formed a halogen bond (or σ-hole interaction) with a water positioned by Ser^292^. Comparison of the apo (PDB ID 6VO7) and liganded structures showed a 2.8 Å shift in the N-terminus of helix 4 and a 2.4 Å shift in the position of the Tyr^246^ side chain (Fig. S1D) (15). Each of these movements is required for the molecule to form the pocket defined by helices 3 and 4. VVD-442 bound on the opposite side of β-strands 1 and 2 of the RBD, relative to the site of the primary interaction with RAS. Thus, we expect that the molecule does not block RAS-p110α binding through direct steric occlusion. Superposition of the RBD VVD-442 complex with the crystal structure of p110α-RAS-glue complex (PDB 9C1F, RMSD 0.70 Å for Cα of residues 151-300) indicated that the inhibitor bound state was incompatible with the RAS bound state (Fig. 1F). Specifically, the positions of Arg^230^, Ser^231^ and Leu^233^ in the VVD-442 bound structure were incompatible with the switch I region of RAS observed in the p110α-RAS-glue complex. This model is consistent with experimental data showing that binding of VVD-442 to p110α disrupts the RAS-p110α interaction (Fig. 1C).

### Cellular characterization of the RAS-p110α blocker VVD-699

**Figure 2:**
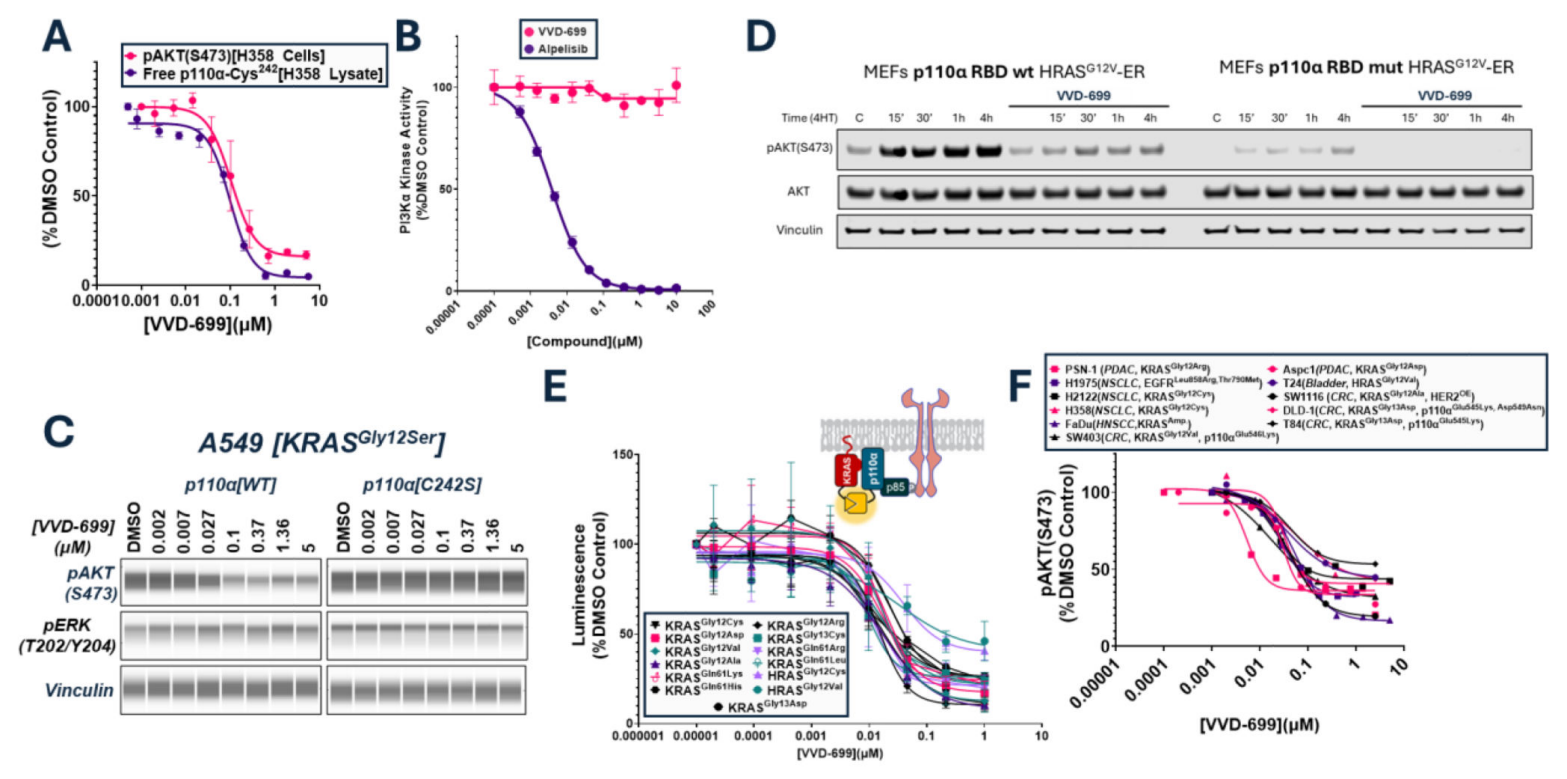
VVD-699 blocks the RAS-p110α interaction in cells and inhibits PI3Kα activation (A) H358 cell lysates treated with VVD-699 for 2 hours to measure binding to Cys^242^ of p110α through pocket probe analysis, a competition-based assay measuring VVD-699’s ability to prevent binding of a biotinylated probe at Cys^242^ of p110α (n=2 biological replicates, SD), and H358 cells treated with VVD-699 for 30 minutes to measure phosphorylated AKT at Ser^473^ by Homogeneous Time-Resolved Fluorescence (HTRF) (n=4 biological replicates, SD). VVD-699 was screened for its ability to inhibit phosphorylation of AKT at Ser^473^ in H358 cells >5 times, the data presented is representative of these experiments. (B) PI3Kα kinase activity assay performed in the presence of VVD-699 or alpelisib (n=2 biological replicates, SD) (C) A549 cells, p110α wild-type or Cys^242^Ser, treated with VVD-699 for 2 hours were collected and activation of the PI3K/AKT pathway(using phosphorylated AKT at Ser^473^) and MAPK pathway (using phosphorylated ERK1/2 at Thr^202^/Tyr^204^) pathways were assessed by Protein Simple western blot. (D) p110α^WT^ or p110α^mut (Thr208Asp/Lys227Ala)^ MEFs were pre-treated 4 hours with DMSO or VVD-699 followed by addition of 100nM 4-hydroxytamoxifen (4HT) to induce expression of HRAS^Gly12Val^. Samples were collected at the indicated timepoints and subjected to western blot analysis. Representative of three independent experiments. (E) VVD-699 was screened for its ability to disrupt the interaction between p110α and various RAS mutants using the NanoBiT protein-protein interaction assay in H358 cells (n=4-12 biological replicates, SD). VVD-699 has been screened against KRAS^Gly12Cys^ >5 times in this assay format, the data presented is representative of these experiments. (F) VVD-699 was screened across numerous cell lines and activation of PI3K/AKT pathway was measured through assessment of phosphorylated AKT at Ser^473^ by HTRF or western blot analysis. VVD-699 was screened in each cell line between 1 and >5 times. In cases where the cell line was screened >1 time, the data presented is representative of these experiments.

To confirm that VVD-699 disrupts the RAS-p110α interaction in cells, we initially focused on KRAS mutant expressing H358 human lung cancer cells, which rely on KRAS^Gly12Cys^ for maximal PI3K activation (16). Using a competition-based assay measuring VVD-699’s ability to prevent binding of a biotinylated probe at Cys^242^ of p110α, we determined that VVD-699 engaged Cys^242^ with a TE_50_ of 98 nM in H358 cell lysates (Fig. S2B for assay design and structure of probe). VVD-699 also inhibited activation of PI3Kα in H358 cells with a half-maximal inhibitory concentration (IC_50_) of 104 nM, based on inhibition of the phosphorylation of Ser^473^ on the protein kinase AKT (hereinafter referred to as phosphorylated AKT), a known downstream target of PI3Kα (Fig. 2A). VVD-699 had no impact on PI3Kα lipid kinase activity in a biochemical assay, supporting the idea that cellular inhibition of phosphorylated AKT did not result from direct inhibition of kinase activity (Fig. 2B). VVD-699 also inhibited phosphorylated AKT in KRAS^Gly12Ser^ A549 human lung cancer cells, and this inhibition was lost in A549 cells that were modified through Clustered Regularly Interspaced Short Palindromic Repeats (CRISPR) technology to express p110α-Cys^242^Ser (Fig. 2C). To further confirm that VVD-699 inhibited RAS promoted p110α activity, we utilized mouse embryonic fibroblasts (MEFs) that were engineered to express a Harvey rat sarcoma virus (HRAS)^Gly12Val^-estrogen receptor (ER) fusion protein, allowing for inducible activation of RAS in cells exposed to the estrogen receptor ligand 4-hydroxytamoxifen (4HT) (17). p110α-WT MEFs treated with VVD-699 before addition of 4HT strongly inhibited HRAS^Gly12Val^ driven activation of PI3Kα (Fig. 2D). In MEFs expressing p110α with blocking mutations in the RBD (Thr^208^Asp and Lys^227^Ala), activation of PI3Kα activity by RAS was also greatly impaired.

We evaluated the effect of VVD-699 on p110α interactions with RAS and the impact on downstream signaling by various RAS mutations that lead to PI3K-AKT pathway activation. In a live cell NanoBiT assay, VVD-699 inhibited the interaction of all tested KRAS and HRAS mutants with p110α with broadly similar potency and magnitude of disruption (Fig. 2E). Interaction of RAS proteins with a p110α-helical domain mutant, measured through NanoBiT, was similarly inhibited (Fig. S2C). Consistent with the NanoBiT findings, VVD-699 also inhibited phosphorylation of AKT in numerous cancer cell lines harboring various RAS mutations, as well as in cell lines with mutations in both KRAS and p110α. Maximum inhibition was variable, but present in all cases (Fig. 2F). These findings show that VVD-699 interferes with the interaction of p110α and RAS through covalent binding of Cys^242^ and inhibits downstream pathway activation across a wide range of cancer relevant RAS and p110α mutations.

### VVD-699 inhibits HER2 activity through an H/K/N-RAS independent mechanism

**Fig. 3:**
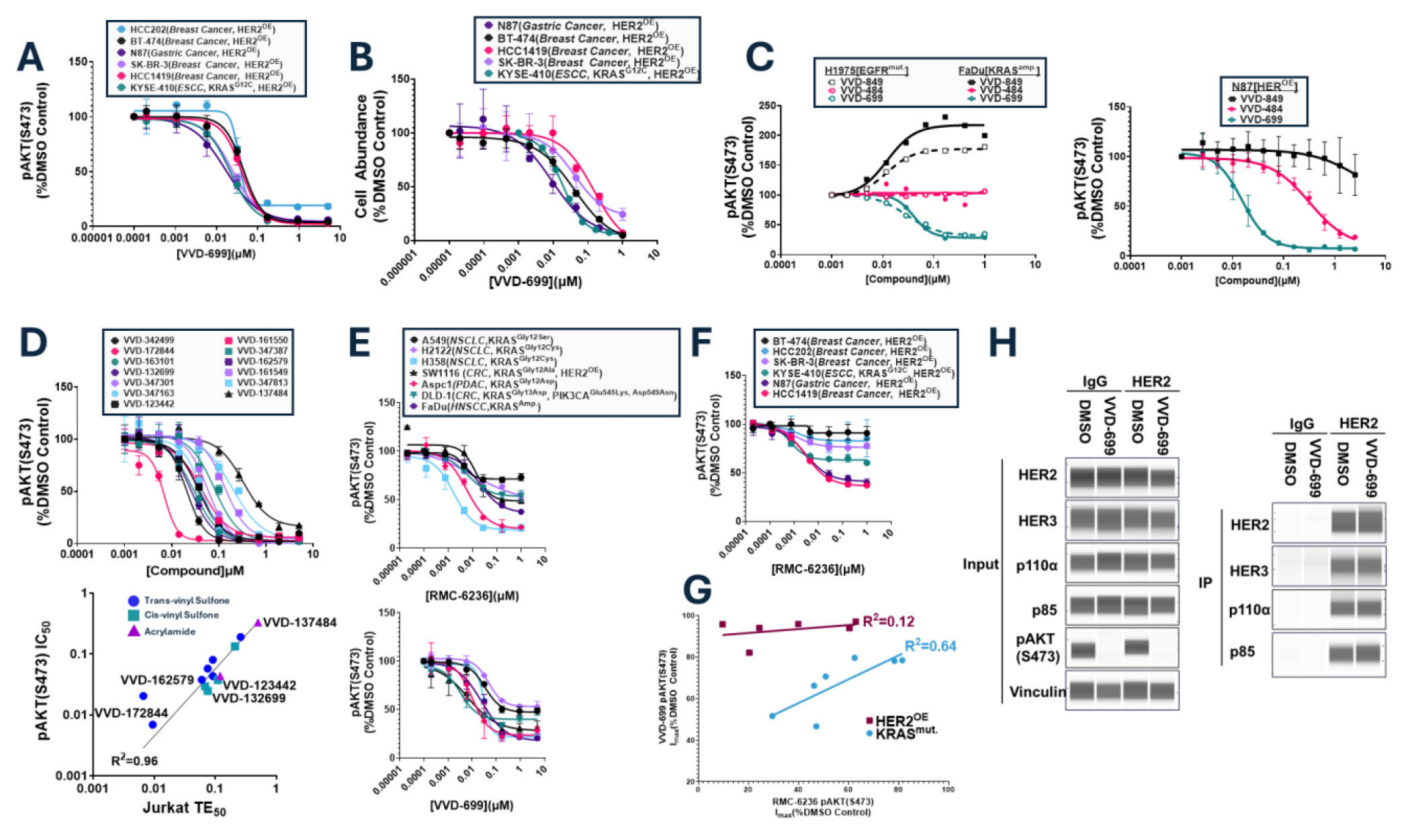
HER2 overexpressing cells show unique sensitivity to VVD-699 (A) HER2 overexpressing cells treated with VVD-699 for 2 hours followed by analysis of phosphorylated AKT at Ser^473^ by HTRF (n=3-6 biological replicates, SD). (B) HER2 overexpressing cells treated with VVD-699 every 3 days for either 6 or 9 days, followed by assessment for cell abundance using Cell Titer Glo (n=1-3 biological replicates, SD). (C) H1975(EGFR^Leu858Arg, Thr790Met^) or FaDu(KRAS^amp^) cells, driven by RAS activity, or N87 cells (n=2 independent experiments, 2-4 biological replicates per experiments, SD), driven by HER2 overexpression, were treated with either VVD-849, VVD-484 or VVD-699 for 2 hours, followed by analysis of phosphorylated AKT at Ser^473^ by HTRF. (D) N87 cells were treated with the indicated compound for 2 hours, followed by analysis of phosphorylated AKT at Ser^473^ by HTRF(Top) (n=2 biological replicates, SD). Linear regression analysis of compound potency of inhibition of phosphorylation of AKT(IC_50_) and compound potency towards binding to p110α-Cys^242^(Bottom). (E) Cells driven by RAS activity (D) or HER2 overexpression (E) were treated with RMC-6236 or VVD-699, followed by analysis of phosphorylated AKT at Ser^473^ by HTRF (n=2-3 biological replicates, SD). (F) Cells driven by HER2 overexpression were treated with RMC-6236 or VVD-699, followed by analysis of phosphorylated AKT at Ser^473^ by HTRF (n=3-6 biological replicates, SD). (G) Linear regression analysis of maximal inhibition seen with RMC-6236 or VVD-699 in KRAS^mut^(Fig. 3E) and HER^OE^(Fig. 3A & 3F) cells. (H) N87 cells were treated with VVD-699 for 2 hours, lysed and then incubated with IgG or a HER2-specific antibody overnight, followed by an hour-long incubation with Protein G magnetic beads. The input and immunoprecipitated samples were analyzed by Protein Simple western blot.

Despite the clear impact of VVD-699 on p110α-RAS interaction and inhibition of downstream signaling, we only observed a small impact on cell growth of KRAS mutant cell lines, including those also containing p110α mutation, in proliferation assays done in anchorage independent settings (Fig. S2D). However, cell lines with overexpression of HER2 protein (HER2^OE^) (18) were particularly sensitive to VVD-699, exhibiting greater inhibition of AKT phosphorylation than in RAS mutant settings, and clear inhibition of cell growth in culture (Fig. 3A-B). To further explore the mechanism of robust HER2 signaling inhibition, we evaluated the glue and silent ligands described above that potently engage p110α-Cys^242^ but differentially impact the p110α-RAS interaction (Fig. 1C and Fig. S1B). In the Epidermal Growth Factor Receptor (EGFR) mutant cell line H1975 (EGFR^Leu858Arg, Thr790Met^) and the KRAS amplified (KRAS^amp^) cell line FaDu, which both show dependence on the interaction of RAS with p110α (19, 20), the impact of the compounds on phosphorylation of AKT (Fig. 3C) was consistent with their behavior in the RAS-p110α NanoBiT assay (Fig. 1C). However, in the HER2 overexpressing cell line N87, the effect of the compounds was not correlated with their disruption of the RAS-p110α interaction as both VVD-699, a blocker, and VVD-484, a silent ligand, strongly inhibited phosphorylation of AKT. We also treated N87 cells with VVD-849, a glue, which partially inhibited phosphorylation of AKT, potentially explained by a stimulatory effect towards the RAS-p110α interaction in addition to suppression of HER2-driven activation of PI3Kα. Additionally, p110α-Cys^242^ ligands representing a variety of chemotypes, and levels of RAS-p110α disruption, all showed near complete inhibition of phosphorylation of AKT in N87 cells, with IC_50_s that strongly agreed with their p110α-Cys^242^ TE_50_s, (R^2^=0.96)(Fig. 3D, Fig. S3C). The disconnect between inhibition of AKT phosphorylation by these ligands in the RAS-dependent vs. HER2-dependent settings suggests that the impact of p110α-Cys^242^ ligands on HER2 signaling may occur predominantly through a RAS-independent mechanism. We treated KRAS mutant and HER2 overexpressing cells with RMC-6236, a small molecule that inhibits activity of all RAS isoforms through recruitment of cyclophilin A, preventing interaction with effector proteins (21). In both settings, RMC-6236 was highly effective at inhibiting the MAPK pathway (Fig. S3A-B). In KRAS mutant cells, we find that RMC-6236 and VVD-699 both partially inhibit phosphorylation of AKT with comparable activity in most cell lines (Fig. 3E). However, in HER2 overexpressing cells, inhibition of phosphorylation of AKT by RMC-6236 was highly variable (Fig. 3F), whereas VVD-699 provided near full inhibition of phosphorylation of AKT (Fig. 3A). Comparison of phosphorylation of AKT inhibition by RMC-6236 and VVD-699 in KRAS mutant cells resulted in good agreement through linear regression analysis (R^2^=0.64) whereas an analogous analysis in HER2 over-expressing (HER2^OE^) cells displayed minimal agreement (R^2^=0.12) (Fig. 3G). The response of HER2 over-expressing cells to VVD-699 was not caused by de-stabilization of p110α, as has been described for the mutant selective p110α inhibitor, Inavolisib, which induces turnover of mutant, but not wild-type, p110α in a HER2-dependent manner (Fig. S3D) (22). Additionally, treatment with VVD-699 did not impact recruitment of PI3Kα to the HER2/3 heterodimer in N87 cells (Fig. 3H). We explored the possibility that p110α-Cys^242^ ligands might prevent HER2 signaling through impacts on non-canonical RAS isoforms that are not efficiently inhibited by RMC-6236 such as MRAS and RRAS. In a live cell NanoBiT assay we found that disruption of the interaction between RAS and p110α through mutation of Thr^208^ and Lys^227^ on p110α (p110α^mut^) disrupted the interaction between all evaluated RAS related proteins and p110α (Fig. S3E). None of the VVD ligands, including VVD-699, strongly inhibited the interaction between p110α and these alternate RAS isoforms. In fact, VVD-484, which inhibited phosphorylation of AKT in N87 cells, functioned either as a silent ligand or an activator when p110α was paired with the alternate RAS isoforms (Fig. S3F). In sum, these data indicate that the activity of p110α-Cys^242^ ligands in the HER2^OE^ setting may reflect a distinct and receptor-specific mechanism that is orthogonal to the molecule’s effect on RAS-p110α interactions.

### VVD-699 Inhibits Tumor Growth Without Impacting Glucose Homeostasis

**Fig. 4:**
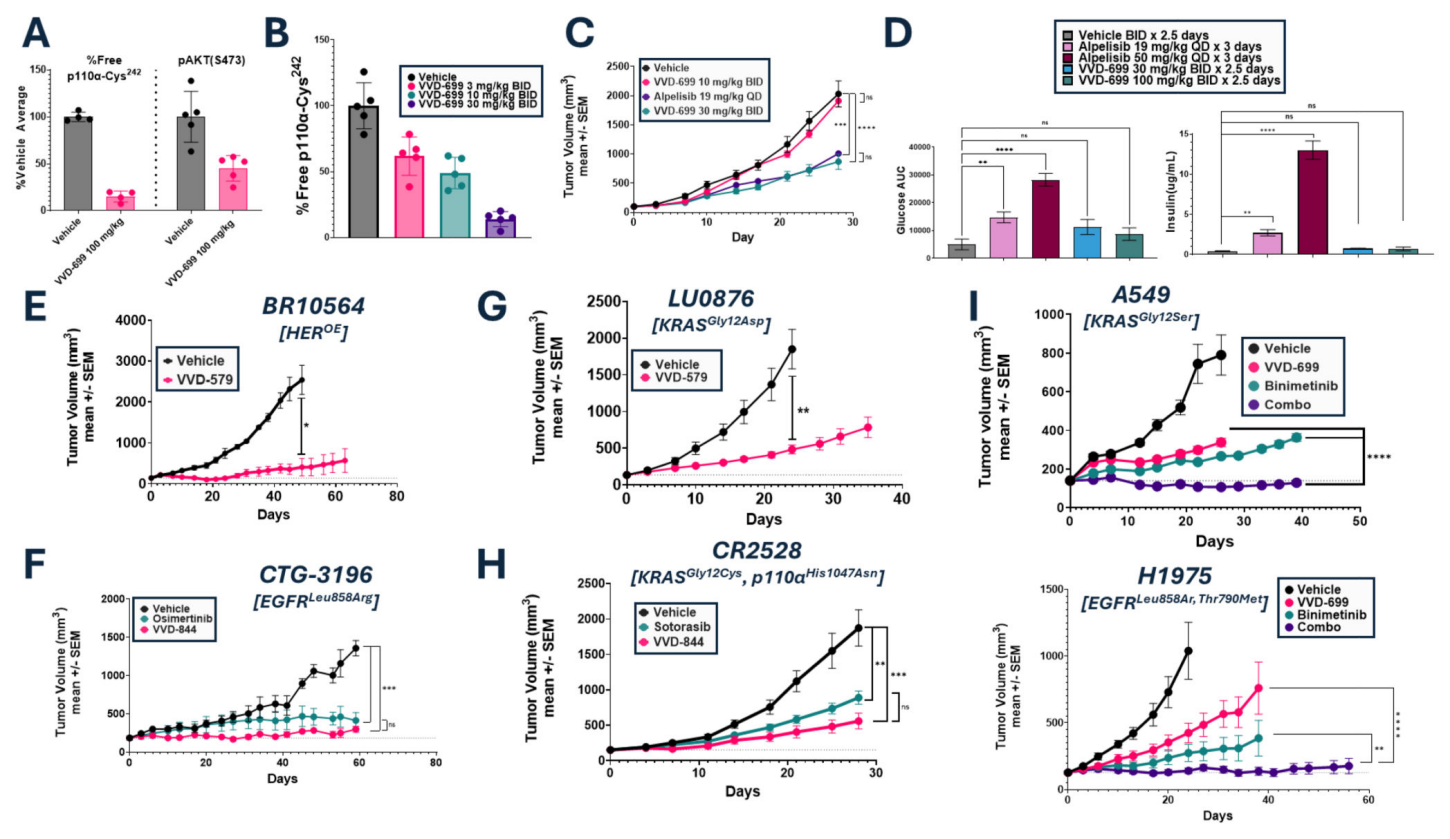
Disruption of the RAS-PI3Kα interaction impairs tumor growth (A) Mice bearing FaDu(KRAS^Amp^) xenografts were given an oral 100 mg/kg dose of VVD-699. 6 hours post-dose, tumor samples were collected and compound binding to Cys^242^ on p110α was determined by mass spectrometry and phosphorylated AKT at Ser^473^ levels were measured via Protein Simple western blotting. (B) Mice bearing FaDu(KRAS^Amp^) xenografts were dosed orally with 3, 30 or 30 mg/kg dose of VVD-699 for 3 days. 6 hours post-dose, tumor samples were collected and compound binding to Cys^242^ on p110α was determined by mass spectrometry. (C) Anti-tumor efficacy of VVD-699 in FaDu(KRAS^amp^) xenografts. Mice were dosed orally with vehicle, 10 mg/kg VVD-699 BID, 30 mg/kg VVD-699 BID or 19 mg/kg alpelisib QD. Data are shown as mean ± SEM; n=10 animals/group for vehicle and alpelisib treated animals, n=9 animals/group for 10 & 30 mg/kg VVD-699 treated animals. Tumor volumes on day 28 were analyzed using a one-way ANOVA with each group compared against each other with a Tukey’s multiple comparison test. ***, p=0.0003, ****, p=<0.0001; ns=not significant. (D) Mice were administered the identified doses of alpelisib or VVD-699 for 3 days, followed by serial measurement of blood glucose levels for 4 hours post-last dose. Blood glucose AUC was then calculated for each treatment group. Additionally, at the conclusion of the time course, plasma was collected and insulin levels were measured. Blood glucose AUC values were analyzed using an ordinary one-way ANOVA test with each treatment group compared against vehicle using a Dunnett’s multiple comparisons test. **, p=0.0089; ****, p=<0.0001; ns= not significant. Plasma insulin values were analyzed using a Kruskal-Wallis test with multiple comparisons. **, p=0.0040; ****, p=<0.0001; ns= not significant. (E) Anti-tumor efficacy of VVD-579 in HER2^OE^ PDX model BR10564. Data are shown as mean ± SEM; n=3 animals/group. Mice were dosed orally BID with 30 mg/kg VVD-579. Tumor volumes on Day 49 were analyzed using a Welch’s t-test, *, p=0.0119. (F) Anti-tumor efficacy of VVD-844 in EGFR^mut^ PDX models CTG-3196. Data are shown as mean ± SEM; n=3 animals/group. Mice were dosed orally BID with 10 mg/kg VVD-844 or orally QD with 25 mg/kg osimertinib. Tumor volumes on Day 59 were analyzed using a one-way ANOVA, with each group compared against each other through a Tukey’s multiple comparison test. VVD-844 vs Vehicle,***, p=0.0003; Osimertinib vs Vehicle, ***, p=0.0006; ns=not significant. (G) Anti-tumor efficacy of VVD-579 in KRAS^Gly12Asp^ PDX model LU0876. Data are shown as mean ± SEM; n=3 animals/group. Mice were dosed orally BID with 30 mg/kg VVD-579. Tumor volumes on Day 29 were analyzed using a Welch’s t-test, **, p=0.0060.(H) Anti-tumor efficacy of VVD-844 in KRAS^Gly12Cys^, p110α^His1047Gln^ PDX model CR2528. Data are shown as mean ± SEM; n=5 animals/group. Mice were dosed orally BID with 10 mg/kg VVD-844 or orally QD with 100 mg/kg sotorasib. Tumor volumes on Day 28 were analyzed using a one-way ANOVA, with each group compared against each other through a Tukey’s multiple comparison test. **, p=0.0038; ***, p=0.0004: ns=not significant. (I) Anti-tumor efficacy of VVD-699, binimetinib, or a combination of both in A549(KRAS^Gly12Ser^) (Top) or H1975(EGFR^Leu858Arg, Thr790Met^) (Bottom) xenografts. Data are shown as mean ± SEM; n=10 animals/group. Mice were dosed orally BID with 30 mg/kg VVD-699, QD with 30 mg/kg binimetinib or a combination of both. Both studies were analyzed using a 2-way ANOVA with each group compared against each other through a Tukey’s multiple comparison test. **, p=0.0068; ****, p=<0.0001. BID, twice daily; QD, once daily; SEM, standard error of the mean; AUC, area under the curve.

Although we did not observe broad impact on RAS driven cell growth of our p110α-RAS blockers *in vitro*, it is possible that the biological importance of the p110α-RAS interaction may be more pronounced *in vivo*. To confirm that VVD-699 is active *in vivo*, mice bearing FaDu xenografts (KRAS^amp^), were given a single 100 mg/kg oral dose of VVD-699. 6 hours later, near maximal binding to Cys^242^ on p110α as well as partial inhibition of AKT phosphorylation were observed (Fig. 4A). Mice carrying FaDu xenografts were dosed orally for 3 days with a range of VVD-699 doses using a twice daily (BID) schedule, followed by assessment of p110α-Cys^242^ binding within the tumor 6 hours after the dose. 86% binding to Cys^242^ on p110α was seen with a 30 mg/kg BID dosing schedule, which was then used for subsequent efficacy studies (Fig. 4B). VVD-699 induced partial tumor growth inhibition comparable to that of a 19 mg/kg oral dosing of alpelisib, a dose that mimics clinical usage (Fig. 4C). Alpelisib is used clinically at doses that minimize the risk of inducing hyperglycemia, a dose limiting toxicity, in patients. All treatment regimens were well-tolerated (Fig. S4A). To evaluate the impact of VVD-699 on glucose handling and insulin production, we measured blood glucose and plasma insulin concentration in mice 3 days after treatment with either alpelisib or VVD-699. Mice treated orally with alpelisib at 19 mg/kg once per day (QD) showed increased insulin production and a modest impact on blood glucose levels. However, the impact on both blood glucose levels and insulin production was dramatically increased by a 50 mg/kg dose of alpelisib (Fig. 4D, Fig. S4B). Neither 30 nor 100 mg/kg of VVD-699, both of which achieved near maximal binding to p110α-Cys^242^ in the spleen and liver, affected blood glucose or plasma insulin levels (Fig. 4D, Fig. S4B). These results highlight that blocking the RAS-p110α interaction without impacting intrinsic kinase activity (Fig. 2B) avoids disruption of glucose control.

To further characterize the breadth of efficacy of p110α–Cys^242^ ligands, we evaluated VVD-699 and alternative chemotypes with similar activity (Fig. S2A, Table S1), in various tumor models. Prior to inclusion in an efficacy study, compounds were profiled in tumor time-course studies at multiple dose levels to characterize target coverage, as measured by binding to Cys^242^ (Fig. S4F). Subsequent dose-range efficacy studies, assessing relative anti-tumor effect and binding to Cys^242^ in the tumor, were performed to confirm that the anti-growth effects observed were consistent with levels of p110α-Cys^242^ binding (Fig. S4G). Growth inhibition was seen in patient derived xenograft (PDX) models in which HER2 is overexpressed (Fig. 4E), consistent with our *in vitro* signaling results. Additionally, VVD-844 inhibited tumor growth in EGFR^mut^ PDX models, including models derived from patients that developed resistance to the third generation EGFR inhibitor, osimertinib (Fig 4F). We also identified sensitivity to these compounds in other KRAS^mut^ PDX models, including models carrying the two most common KRAS mutations (23), Gly^12^Asp (Fig. 4G) and Gly^12^Val (Fig. S4C). VVD-844 also suppressed growth of a PDX model carrying co-mutation of both KRAS and p110α, in this case providing comparable activity to direct targeting of KRAS^Gly12Cys^ (Fig. 4H). These data highlight the importance of the RAS-p110α interaction across a variety of backgrounds; however, the lack of complete tumor growth inhibition supports observations that targeting individual RAS effector pathways results in limited efficacy (24). With this in mind, we explored disruption of the RAS-p110α interaction in combination with a MEK inhibitor, to simultaneously inhibit the PI3K and MAPK pathways. A549 (KRAS^Gly12Ser^) cells treated *in vitro* with VVD-699 and the MEK inhibitor, binimetinib, showed inhibition of both the PI3K/AKT and MAPK pathways (Fig. S4D). We tested this combination in mice with A549 and H1975 xenografts (Fig. 4I). In both models, the combination treatment generated a significantly improved response relative to either single agent, with durable tumor stasis observed. Interestingly, although H1975 was far more responsive than A549 to VVD-699 in 3D proliferation assays (Fig. S2D), similar efficacy is seen *in vivo*, highlighting the challenge of assessing sensitivity to this mechanism of action *in vitro*. We evaluated the inhibitor combination in KRAS^mut^ PDX models, finding benefit relative to the individual therapies across models carrying various mutations to KRAS (Supp. Fig. 4E).

### Blocking the RAS-p110α interaction sensitizes tumors to KRAS^Gly12Cys^ Inhibition

**Fig. 5:**
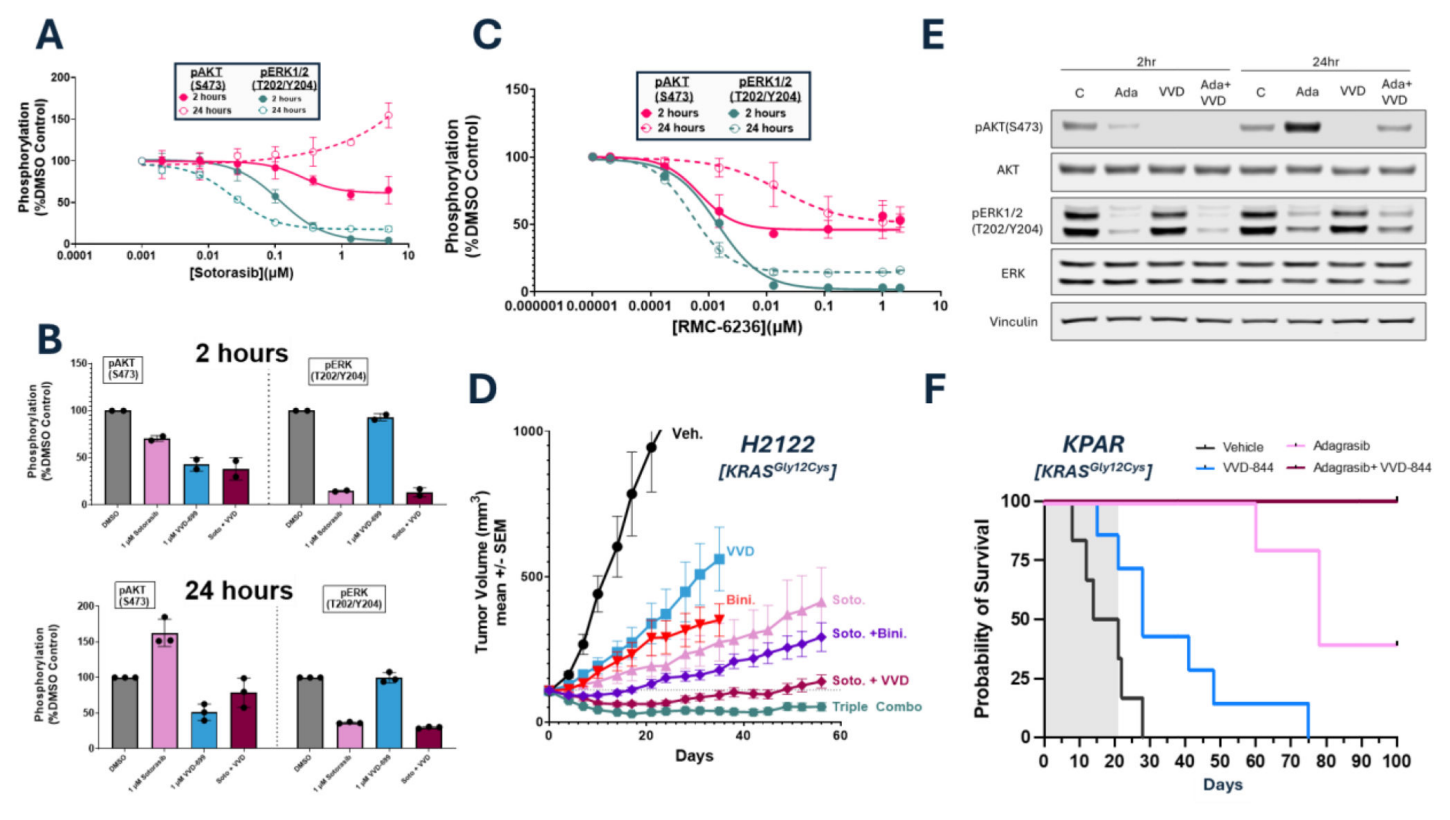
Blocking the RAS-p110α interaction sensitizes tumors to KRAS^Gly12Cys^ Inhibition (A) H2122 cells treated with sotorasib for 2 or 24 hours. At each timepoint, cells were harvested and activation of the PI3K/AKT (using phosphorylated AKT at Ser^473^) and MAPK (using phosphorylated ERK1/2 at Thr^202^/Tyr^204^) pathways were assessed by western blot (n=3 biological replicates, SD. Sotorasib has been screened in this format, or a similar one, in H2122 cells >5 times, the data presented is representative of these experiments). (B) H2122 cells were treated with the stated dose of sotorasib, VVD-699 or a combination of both for 2 or 24 hours. At each timepoint, cells were harvested and activation of the PI3K/AKT (using phosphorylated AKT at Ser^473^) and MAPK (using phosphorylated ERK1/2 at Thr^202^/Tyr^204^) pathways were assessed by Protein Simple western blot (n=2 biological replicates at 2 hours and n=3 biological replicates at 24 hours, SD. This assay has been performed >5 times, the data presented is representative of these experiments). (C) H2122 cells treated with RMC-6236 for 2 or 24 hours. At each timepoint, cells were harvested and activation of the PI3K/AKT (using phosphorylated AKT at Ser^473^) and MAPK (using phosphorylated ERK1/2 at Thr^202^/Tyr^204^) pathways were assessed by Protein Simple western blot (n=2 biological replicates, SD). (D) Anti-tumor efficacy of VVD-699, sotorasib, binimetinib, or varying combination of both in H2122(KRAS^Gly12Cys^) xenografts. Data are shown as mean ± SEM; n=10 animals/group. Mice were dosed orally BID with 30 mg/kg VVD-699, QD with 30 mg/kg binimetinib or QD with 100 mg/kg sotorasib. The same doses of each individual therapy were maintained when used in combination. (E) *In vitro* KPAR^Gly12Cys^ mouse cells were treated with 100 nM adagrasib, 10 nM VVD-844 or a combination of both for 2 or 24 hours. Cells were harvested and activation of the PI3K/AKT (using phosphorylated AKT at Ser^473^) and MAPK (using phosphorylated ERK1/2 at Thr^202^/Tyr^204^) pathways were assessed by western blot. (F) Survival of mice bearing KPAR^Gly12Cys^ orthotopic lung tumor treated with vehicle (n=6), 50 mg/kg QD adagrasib (n=5), 10 mg/kg QD VVD-844 (n=7) or the combination (n=5) for three weeks (grey area). BID, twice daily; QD, once daily; SEM, standard error of the mean.

Although patients initially respond to KRAS^Gly12Cys^ inhibitors, tumors quickly develop resistance to the therapy (25). Post-treatment analysis indicates that this occurs through both genetic and non-genetic mechanisms of resistance, nearly all of which lead to reactivation of the RAS signaling pathways (26). We focused on H2122 cells, which are intrinsically partially resistant to KRAS^Gly12Cys^ inhibition (27). When these cells were treated with the KRAS^Gly12Cys^ inhibitor, sotorasib, for 2 hours, both the MAPK and PI3K/AKT pathways were effectively suppressed, highlighting the role of KRAS^Gly12Cys^ in activation of these pathways (Fig. 5A). However, after 24 hours of sotorasib treatment, although MAPK pathway inhibition was maintained, signaling through the PI3K/AKT pathway was restored. In contrast, VVD-699 maintained inhibition of AKT phosphorylation in H2122 cells at both 2 and 24 hours (Fig. S5A). Combination treatment with VVD-699 and sotorasib suppressed both PI3K/AKT and ERK signaling at 2 and 24 hours, indicating that this combination can be used to more durably suppress the PI3K/AKT and MAPK pathways in KRAS^Gly12Cys^ driven cancers (Fig. 5B, Fig. S5B). As wild-type RAS isoforms support signaling in KRAS^mut^ cancers (28), we evaluated the role of wild-type HRAS or Neuroblastoma rat sarcoma virus (NRAS) in H2122 cells, which are homozygous for KRAS^Gly12Cys^. CRISPR mediated deletion of HRAS or NRAS in H2122 cells led to a decrease in phosphorylation of AKT, but not of phosphorylated ERK (Thr^202^/Tyr^204^) (Fig. S5C). These results indicate that while activation of the MAPK pathway is dominantly controlled by KRAS^Gly12Cys^ in H2122 cells, activation of the PI3K/AKT pathway is more complex and has contributions from wild-type RAS isoforms. Additionally, as wild-type HRAS and NRAS function in the basal signaling network of H2122 cells, they may also help drive the rebound in PI3K/AKT signaling seen with prolonged inactivation of KRAS^Gly12Cys^. In support of this, RMC-6236 maintained pAKT inhibition at 24 hours treatment, albeit it with decreased potency (Fig. 5C).

To test the relevance of these *in vitro* findings, we performed an *in vivo* efficacy study using H2122 xenografts, testing VVD-699 or the MEK inhibitor, binimetinib, in combination with sotorasib (Fig. 5D). VVD-699 provided a more profound benefit in combination with sotorasib than binimetinib combined with sotorasib, supporting our *in vitro* observations that sotorasib more effectively inhibits the MAPK pathway than the PI3K/AKT pathway. Although tumors treated with these inhibitor combinations showed outgrowth, the three drug combination of VVD-699, sotorasib and binimetinib showed strong and durable tumor regression. We also evaluated our RAS-p110α blockers alone and in combination with the KRAS^Gly12Cys^ inhibitor, adagrasib, in the KPAR(KRAS^Gly12Cys^) mouse lung cancer cell line (29). The *in vitro* behavior of adagrasib, and VVD-844, alone and in combination was consistent with our observations in H2122 cells, indicating that WT RAS dependent re-activation of the PI3K pathway is a common response to KRAS^Gly12Cys^ inactivation (Fig. 5E). In immune competent mice, the growth of subcutaneously implanted KPAR tumors was impaired by treatment of the RAS-p110α blocker VVD-844 (Fig. S5D).To assess the role of the RAS-p110α interaction in a setting that more faithfully reflects the tumor microenvironment, immune competent mice bearing KPAR orthotopically implanted lung tumors were treated with either adagrasib, VVD-844, or a combination for three weeks, followed by discontinuation of dosing and close health monitoring until humane endpoint reached. VVD-844 alone had little impact on survival, while adagrasib treatment led to 40% of the mice surviving more than 90 days. However, the combination of VVD-844 and adagrasib was more effective, with 100% of the mice showing complete and durable responses (Fig. 5F).

## Discussion

Our results emphasize the importance of the interaction between RAS and the p110α RBD in the control of PI3Kα activity in cancer cells. Molecules such as VVD-699 and VVD-844 block the interaction between RAS and PI3Kα, and inhibit PI3Kα activity and tumor growth in a wide range of RAS dependent cancer models. These findings confirm prior results from genetic models (6,7) demonstrating the importance of the RAS-PI3Kα interaction to support RAS driven tumorigenesis. The strong effect of our Cys^242^ ligands on HER2 driven PI3Kα signaling and HER2 dependent tumor growth, by contrast is surprising and unexpected. These findings, as well as those presented in the RAS driven setting, are consistent with the recently published characterization of BBO-10203 (34), which also inhibits the RAS-PI3Kα interaction through covalent binding to Cys^242^. VVD-699 and BBO-10203 are structurally distinct molecules, reflective of unique paths to discovery, leading to differing mechanisms by which they disrupt the RAS-PI3Kα interaction– VVD-699 binds adjacent to the RAS-p110α interface and stabilizes a conformation of the p110α RBD that is incompatible with RAS binding, whereas BBO-10203 induces a direct steric clash at the interface between RAS and p110α. Despite these differences both molecules have strikingly similar impacts on PI3Kα signaling across a range of cell types including both RAS mutant and HER2 overexpressing models and inhibit tumor growth *in vivo* without inducing hyperglycemia.

The finding that HER2 overexpressing cancers are particularly sensitive to modification of Cys^242^ of PI3Kα by the molecules described here and also BBO-10203 highlights the surprising potential for this strategy to benefit patients whose disease is driven by this signaling pathway. In their manuscript, Simansu et. al propose that BBO-10203 is likely to inhibit HER2 signaling through preventing PI3Kα interaction with a non-canonical RAS isoform. This proposal is inconsistent with our observation that VVD-484, which binds to Cys^242^ but does not block the RAS-PI3Kα interaction, is highly effective at blocking HER2 signaling through PI3Kα. We further determined that VVD-484 does not prevent the interaction of PI3Kα with multiple non-canonical RAS isoforms. The mechanism through with the p110α RBD, and specifically Cys^242^, is involved in HER2 signaling to PI3Kα remains unclear and will require further exploration.

Amongst the Cys^242^ targeting compounds discovered with our chemoproteomics platform, we identified not only inhibitors of the RAS-PI3Kα interaction, but also inducers of the interaction that increase PI3Kα activity, and neutral binders have no impact on the interaction or activity. This is reminiscent of work reporting that different mutations at a single residue in the RBD of p110α can either inhibit (Lys^227^Ala) or activate (Lys^227^Glu) PI3Kα activity (31), as well as two studies published while this manuscript was under review detailing molecules that bind to the same Cys^242^ adjacent pocket as our covalent molecules and either stabilize (35), or block (34) the RAS-p110α interaction. Although inhibitors and their possible role in cancer therapy were our main focus, inducers of the RAS-PI3Kα interaction could also have clinical utility for activating PI3K downstream of receptor tyrosine kinases, for example as a way of promoting tissue regeneration or treating insulin-resistant diabetes (32, 33).

While our work confirms prior studies in GEMMs (6,7) demonstrating that disruption of the RAS-PI3Kα interaction impairs growth of KRAS^mut.^ cancers *in vivo*, it is notable that this sensitivity is not readily observed using *in vitro* proliferation assays for both the compounds presented here as well as BBO-10203. The reason for this discrepancy is currently unclear, however, a tumor extrinsic role for the RAS-PI3Kα interaction has been reported (36), including in angiogenesis, and could provide an explanation. This observation highlights the limitations of *in vitro* cell growth assays when assessing cancer dependencies and evaluating targets as potential therapeutics.

With the recent explosion of clinical trials evaluating RAS targeted therapies, it has become apparent that combination strategies will be necessary to most effectively treat these cancers. The data presented here, as well as that published for BBO-10203 (34), support the idea that targeting the RAS-PI3Kα interaction represents a promising combination approach with multiple approved and investigational agents. Both manuscripts report the potential for combination with KRAS^Gly12Cys^ inhibition. Here, we provide evidence implicating WT H/NRAS in feedback mediated re-activation of PI3Kα in the KRAS^Gly12Cys^ mutant setting, which is effectively inhibited by RAS-PI3Kα blockade but evades mutant selective KRAS inhibitors. This finding highlights the complexity of RAS’ role in PI3K activation as compared to the MAPK pathway, which appears to be dominantly controlled by KRAS. We also found promising combination benefit of RAS-PI3Kα inhibition with MEK inhibition, an approach that could potentially benefit patients with RAS driven cancers regardless of their specific RAS mutation. It is worth noting that Simansu et al. additionally demonstrated combination benefit for BBO-10203 with standard of care agents (trastuzumab, fulvestrant/ribociclib) in the setting of ER positive breast cancer, further highlighting the broad clinical potential of the RAS-PI3Kα targeting strategy.

Activation of PI3Kα through direct mutation or by mutation or overexpression of upstream signaling factors (e.g. KRAS, EGFR, HER2) is one of the most common oncogenic events in human cancers. To date, the clinical utility of PI3Kα selective kinase inhibitory drugs has been limited by a narrow therapeutic window. The discovery of molecules selectively targeting RAS and HER2 mediated activation of PI3Kα provides a promising new approach to more broadly impact patients with PI3Kα activated cancers. The work presented here has led to the initiation of a clinical trial (NCT06804824) evaluating a further optimized molecule (VVD-159642) in patients with RAS pathway activation and HER2 overexpressing cancers.

## Supplementary Material

Supplementary Material

## Data Availability

Atomic coordinates and structure factors for the x-ray crystallographic structure presented have been deposited in the Protein Data Bank with accession code 9E8M,
