# Supplementary material for: Covalent inhibitors of the PI3Kα RAS binding domain impair tumor growth driven by RAS and HER2
Source: Science. Author manuscript; Available in PMC 2025 Oct 29. (PMC7618302; doi:10.1126/science.adv2684)
Supplement: Supplementary Material [file EMS209649-supplement-Supplementary_Material.pdf]

Supplementary Materials for

Covalent inhibitors of the PI3Kα RAS binding domain impair tumor growth driven by RAS and HER2

Joseph E. Klebba[1], Nilotpal Roy[1], Steffen M. Bernard[1], Stephanie Grabow[1], Melissa A. Hoffman[1], Hui Miao[1], Junko Tamiya[1], Jinwei Wang[1], Cynthia Berry[1], Antonio Esparza-Oros[1], Richard Lin[1], Yongsheng Liu[1], Marie Pariollaud[1], Holly Parker[1], Igor Mochalkin[1], Sareena Rana[2], Aaron N. Snead[1], Eric J. Walton[1], Taylor E. Wyrick[1], Erick Aitichson[1], Karl Bedke[1], Jacyln C. Brannon[3], Joel M. Chick[4], Kenneth Hee[5], Benjamin D. Horning[1], Mohamed Ismail[2], Kelsey N. Lamb[1], Wei Lin[1], Justine Lu[1], Martha K. Pastuszka[1], Jonathan Pollock[1], John J. Sigler[1], Mona Tomaschko[2], Eileen Tran[6], Chanyu Yue[1], Todd M. Kinsella[7], Miriam Molina-Arcas[2], Brian N. Cook[1], Gabriel M. Simon[1], David S. Weinstein[1], Julian Downward[2,8], Matthew P. Patricelli[1,8]

Affiliations:

[1]Vividion Therapeutics, 5820 Nancy Ridge Drive, San Diego, California, 92121.

[2]Francis Crick Institute, 1 Midland Road, London NW1 1AT, UK.

[3]Current address: Odyssey Therapeutics, San Diego, CA, USA.

[4]Current address: Self.

[5]Current address: Sciex, Carlsbad, CA, USA.

[6]Current address: Novartis Institutes for Biomedical Research, San Diego, CA, USA.

[7]Current address: Independent researcher, San Diego, CA, USA.

[8]Corresponding author; Email: Julian.Downward@crick.ac.uk, mattp@vividion.com

This PDF file includes:

Materials and Methods

Figs. S1-S5

Tables S1-2

References(*34-35*)

Materials and Methods:

Cell Lines and Culture

Cell line source:

Jurkat (ATCC; catalogue # TIB-152)
HEK293T (ATCC; catalogue # CRL-3216)
H358 (ATCC, catalogue # CRL-5807)
A549 (ATCC, catalogue # CCL-185)
AsPC-1(ATCC, catalogue # CRL-1682)
T24(ATCC, catalogue # HTB-4)
SW1116(ATCC, catalogue #CCL-233)
H1975(ATCC, catalogue # CRL-5908)
H2122(ATCC, catalogue # CRL-5985)
T84(ATCC, catalogue # CCL-248)
SW403(ATCC, catalogue # CCL-230)
DLD-1(ATCC, catalogue # CCL-221)
FaDu(ATCC, catalogue # HTB-43)
HCC-202(ATCC, catalogue # CRL-2316)
BT-474(ATCC, catalogue # HTB-20)
N87(ATCC, catalogue # CRL-5822)
SK-BR-3(ATCC, catalogue # HTB-30)
HCC1419(ATCC, catalogue # CRL-2326)
KYSE-410(Sigma Aldrich, catalogue #94072023-VL)
KPAR1.3 $KRAS^{G12C}$ cells (herein $KPAR^{G12C}$) were generated as previously described (*29*).
Available from CancerTools (https://cancertools.org/cell-lines/kpar-g12c-161804/)
MEFs p110α RBD wt $HRAS^{G12V}$-ER and MEFs p110α RBD mut $HRAS^{G12V}$-ER were generated as previously described *(7)*

Cell Culture conditions:
Cell lines were maintained according to the vendor handling instructions

Compound treatment

Cells were dosed with the indicated dose response or set dose of compound using an HP Digital Dispenser.

For in vivo studies, the following vehicles were used for the respective compounds:

Compound	Vehicle	Associated Figure(s)
VVD-699	10% NMP/90% Labrafil	4A/B/C, S4A
	20% HPβCD+ 0.1% Tween 20	4D, S4B
	0.5%(w/v) CMC + 0.1%(w/v) Tween 20	4I, 5D
VVD-579	20% HPβCD+ 0.1% Tween 20	4E/G, S4C, E(top)F,G
	0.5%(w/v) CMC + 0.1%(w/v) Tween 20	S4E(bottom)
VVD-844	0.5% HPMC in sterile water	4F

	10% Ethanol + 60% PEG 400 +30% DI Water (v:v:v)	4H
Alpelisib	10% NMP/90% Labrafil	4A
	20% HPβCD+ 0.1% Tween 20	4D
Sotorasib	10% Ethanol + 60% PEG 400 +30% DI Water (v:v:v)	4H
	0.5%(w/v) CMC + 0.1%(w/v) Tween 20	5D
Binimetinib	0.5%(w/v) CMC + 0.1%(w/v) Tween 20	4I, 5D, S4E(bottom)
	20% HPβCD+ 0.1% Tween 20	S4E(top)
Osimertinib	0.5% HPMC in sterile water	4F
Adagrasib	10% captisol in 50 mM citrate buffer pH 5	5F, S5D

Targeted Chemoproteomics

Chemoproteomics samples were prepared as previously described and detailed briefly below *(13)*.

<u>In Vitro target engagement</u>

Jurkat or Karpas-299 cell pellets were lysed by probe sonication in Dulbecco's phosphate buffered saline (D-PBS) and, following protein concentration normalization, 500 µg of total protein per well was aliquoted into a 96-well plate. Lysates were incubated with compound for 1 h followed by iodoacetamide-desthiobiotin (IA-DTB) probe-labeling of all solvent exposed cysteines. Protein was acetone precipitated then resuspended in freshly prepared 9M urea with 50 mM ammonium bicarbonate. Proteins were reduced and alkylated by the addition of DTT and iodoacetamide, buffer exchanged with Zeba de-salting plates, then digested with trypsin for 1h. IA-DTB labeled peptides were isolated with streptavidin agarose resin and enriched cysteine-containing peptides were eluted by the addition of 50% acetonitrile (ACN) with 0.1% Formic Acid (FA) and dried until ready for analysis. For VVD-849, probe labeled samples were TMT labeled (detailed below) and target engagement quantified by targeted TMT, as previously described. (*34*)

<u>In Vivo target engagement</u>

Snap-frozen tissues in bead-beater tubes were placed on ice and homogenized in cold Pierce RIPA lysis buffer with 30 s of bead beating at 4 °C. Samples were sonicated and cleared by centrifugation, protein was normalized, and 300 µg per sample aliquoted into a 96-well plate. Samples were prepared as described above, starting with IA-DTB probe-labeling.

<u>Liquid chromatography – tandem mass spectrometry analysis</u>

Probe-labeled peptides were concentrated onto an Acclaim PepMap100 C18 loading column (Thermo, DX164564, 100 µm x 2 cm, 5 µm particle size) and separated on a custom made C18 nanoviper analytical column (Thermo, 75 µm x 15 cm, 2 µm particle size) using a Dionex Ultimate 3000 nano-LC (Thermo). For in vitro experiments, peptides were separated using an

8.7 min gradient going from 6 to 32.5% B solvent (96.4% ACN, 3.5% dimethylsulfoxide (Pierce, PN 20688), 0.1% FA) mixed with A solvent (96.4% water, 3.5% dimethylsulfoxide, 0.1% FA). In vivo samples were separated using the same gradient, but over 12.7 min.

Peptides were analyzed by parallel reaction monitoring (PRM) mass spectrometry using product ion scan mode on the Thermo Exploris 120 orbitrap mass spectrometer in positive ion mode with a spray voltage of 2500V and 375 °C ion transfer tube temperature. Precursor ions were fragmented and measured using a normalized collision energy of 25%, 0.7 m/z Q1 resolution, 30,000 orbitrap resolution, and 70% RF lens. In vitro datasets were acquired with a 200% normalized AGC for all peptides and in vivo datasets were acquired with custom normalized AGC up to 3000% for p110α peptides. Data was acquired using a scheduled method with 1.2 min windows and dynamic injection time mode with a minimum of seven points across the peak.

Parallel Reaction Monitoring (PRM) data analysis

Target engagement (p110α-Cys^{242}) was measured by monitoring peptide LCVLEYQGK (688.8839 m/z) and additional p110α peptides were monitored to ensure target engagement rather than a protein level change. Retention time standard (RTS) peptides were also included in the method and were used for global normalization for each sample. Mass Spectrometry data was analyzed using Skyline v.22.2.0.255 (MacCoss Lab, University of Washington). Peptide quantification was performed by summing the peak areas corresponding to four to six fragment ions. Fragment ions were pre-selected from an in-house generated reference spectral library. RTS peptides were used to normalize for sample variability. Percent target engagement for each peptide was determined by comparing the average peptide AUC of the compound treated group to the average peptide AUC of the control (DMSO or vehicle treated) group.

Global Proteomic Selectivity Profiling

Live cell target engagement

Five million Jurkat cells (in RPMI with 10% FBS) per well were arrayed into a 96-well plate and treated with compound for 2 h. Following compound wash out, cells were lysed by sonication in phosphate buffered saline (PBS) and incubated with IA-DTB for one hour at room temperature. Cysteine peptide enrichment was performed as described above. The resulting dried peptides were resuspended in 0.2 M EPPS pH 8.5 in 30% anhydrous ACN and labeled with 55 µg TMTpro 18plex reagent (Thermo, A52045). Samples were quenched with hydroxylamine. The combined samples were desalted on a Biotage evolute express ABN plate and separated by high pH reversed phase fractionation. 96 fractions were recombined to 12 fractions and analyzed by LC-MS/MS. Three 18-plexes were used to generate this data and compounds were paired as follows: VVD-699/VVD-844, VVD-442/VVD-579, and VVD-484/VVD-849.

Liquid chromatography – tandem mass spectrometry analysis

Cysteine enriched peptides were loaded onto an Acclaim PepMap100 C18 loading column (listed above) and separated on an Acclaim PepMap C18 analytical column (Thermo, 75 µm x 25 cm, 2 µm particle size) using a Dionex Ultimate 3000 nano-LC (Thermo). Separation was using an 83.5 min gradient from 6 to 30% solvent B for VVD-442, VVD-699, VVD-844, and VVD-579 and a 171 min gradient from 6 to 30% solvent B for VVD-484 and VVD-849. Global TMT data was acquired on an Orbitrap Fusion Lumos Tribrid MS (Thermo) using the SPS MS3 workflow.

Global TMT data analysis

Data was searched with MassPike software package (GFY development team, GFY Core Version 3.4) using the Human FASTA database with a precursor mass tolerance of 30 ppm and a fragment ion tolerance of 0.3 m/z and filtered using a 2% false discovery rate. Peptide spectral match results with an XCorr > 1 and a summed DMSO TMT signal > 20 were converted to cysteine site level data, then filtered to remove sites with >40% coefficient of variation (CV) in the DMSO control samples. Each compound was prepared with a dose response above the selected concentration (~25-fold over average IC_{50}s) and sites that did not exhibit a dose response were filtered from the dataset (~300 out of 30,000 sites filtered from VVD-442, VVD-699, VVD-844, and VVD-579 datasets and ~30 out of 20,000 sites filtered from the VVD-484 and VVD-849 dataset). Global data normalization was applied and ratio of average DMSO AUC to compound treated AUC calculated to generate selectivity data plots.

Pocket Probe

p110α-Cys^{242} Pocket Probe Generation

A covalent ligand that binds potently and selectively to Cys^{242} of p110α was identified. An exit vector from this covalent ligand was identified and linked to D-Biotin to generate a p110α-Cys^{242} biotin probe that was used under the assay conditions. This probe has a proteomic TE_{50} of 100 nM in Jurkat cell lysate.

Pocket probe p110α-Cys^{242} target engagement in Jurkat and H358 cell lysates

Cell pellets were thawed and diluted in cold Dulbecco's phosphate buffered saline to a concentration of 300×10^6/12 mL (Jurkat) or 300×10^6/20 mL(H358). Cells were lysed via sonication, and lysate was plated (50 µL/well) in a 96-well plate. The cell lysates were then treated with compound using the HP Digital Dispenser and allowed to incubate at room temperature for 1 hour (Jurkat) or 2 hours (H358). Next, the biotin-conjugated pocket probe was added to each well and allowed to incubate for an additional hour at room temperature. 75 µL of dilution buffer was added to each well, followed by centrifugation and storage at 80°C. On the day of the assay, the lysate was thawed and added to a 96-well plate pre-coated with capture antibody. Following a 1.5 hour incubation, the plate was washed with 100 µL/well PBST, and 20 µL/well of NeutrAvidin-HRP was added and incubated for 1 hour at room temperature. The plate was then washed 4 times with PBST, 100 µL/well. HRPs ubstrate, 20 µL/well, was added to the plate prior to the CLARIOstar Plus luminescence read.

<u>Analysis</u>

To account for non-specific assay signal, first a qualitative assessment was performed to assess stability of the bottom of the curve, i.e. no additional loss in signal at the three highest doses. If so, this residual signal was deemed to be the floor of the assay and the signal at the highest dose of compound, 50 µM, was subtracted from all other datapoints. The treatment samples were then normalized to the DMSO treated samples and the TE_{50} was determined using GraphPad Prism software (part of Dotmatics, Boston MA).

RAS-p110α NanoBiT

<u>HEK293T Lysate format</u>

HEK293T cells plated in full media in a T225 flask were transfected with pBiT2.1-N [TK/SmBiT]_KRAS^{mut}, pBiT1.1-N [TK/gBiT]_p110α ^{wt}/^{Glu545Lys} using Lipofectamine 3000. HSV-TK-p85α was also co-transfected where indicated.

The cells then incubated for 48 hours at 37˚C with 5% CO_2 . The cells were then harvested and lysed through probe sonication, followed by storage of the cell lysate at -80˚C.

On the day of the assay, the lysate was thawed and 20 µL/well of lysate was distributed to a 384 well plate, as well as 20 uL of untransfected HEK293T cell lysates, which served as a negative control. The plate was then treated with compound using the HP Digital Dispenser and following a 1-hour(p85α not co-transfected) or 4-hour(p85α co-transfected) incubation at RT on an orbital shaker, 4 µL of Nano-Glo reagent was added to each well and luminescence was read using the CLARIOstar Plus (BMG LABTECH, North Carolina).

<u>H358 Live Cell Format</u>

To generate the pBiT2.1-CMV-Smbit-RAS expression plasmids, full length cDNA for amplified for the respective isoforms was using PCR and cloned in-frame into the pBiT2.1-N [TK/SmBiT] vector. To generate the pBiT1.1-CMV-Lgbit- p110α WT expression plasmid, p110α was amplified using PCR and cloned in-frame into the pBiT1.1-N [TK/LgBiT] vector. For both expression plasmids, Gibson cloning was utilized to replace the TK promoter with the CMV promoter. To generate pBiT1.1-CMV-Lgbit- p110α RBD, in which p110α can no longer interact with RAS, two point mutations on the RAS binding domain(RBD) of p110α (Thr^{208}Asp and Lys^{227}Ala) were introduced using the Q5 site-directed mutagenesis kit (*7*).

20,000 H358 cells/well were plated in full media in a 96 well plate and incubated overnight at 37˚C with 5% CO_2 . The following morning, the cells were transfected with pBiT2.1-CMV-Smbit-KRAS Gly^{12}Cys(50ng/well), pCMV6-p85β (100ng/well) and pBiT1.1-CMV-Lgbit-p110α ^{WT} or pBiT1.1-CMV-Lgbit- p110α $^{mut.}$ (100ng/well) using Lipofectamine 3000.
The transfected cells were then incubated for 24 hours at 37 °C with 5% $CO_{2.}$ Following this incubation, cell media was removed and replaced with Opti-MEM media, followed by compound treatment using the HP Digital Dispenser. The plate then incubated at 37 °C with 5% CO_2 for 2 hours. Following this incubation, Nano-Glo Live substrate was diluted 1:20 in Nano-

Glo LCS Buffer, pre-warmed to 37 °C. 25 µl of the mixture was added to each well and gently mixed by hand. The plate was then incubated for 20 minutes at 37ºC and then luminescence was determined us the Clariostar Plus plate reader (BMG LABTECH, North Carolina, USA).

pAKT(S473) and pERK1/2(T202/Y204) Homogeneous Time Resolved Fluorescence (HTRF) assays

Cells were harvested, centrifuged at 1000 rpm for 5 minutes and resuspended in media containing 1% FBS. 100 µL of cell suspension was plated into each well of a 96 well plate, corresponding to 25000-40000 cells/well, optimized on a per cell line basis. The plate was incubated overnight at 37 °C with 5% CO_2 to allow the cells to adhere to the plate. The following day, the cells were treated with a dose response of compound. The plate was incubated for the indicated amount of time at 37 °C with 5% CO_2. Following this incubation, media was removed and HTRF lysis buffer was added to each well, followed by an incubation for 45-60 minutes at room temperature. The lysate was then transferred to an HTRF plate pre-loaded with HTRF antibody mix. Additionally, wells were loaded with lysis buffer only and HTRF antibody mix as a negative control. The plate was then sealed and allowed to incubate overnight at room temperature. The following morning, fluorescence intensity was measured on the Clariostar Plus plate reader Company (BMG LABTECH, North Carolina, USA).

The ratio of acceptor/donor fluorescence of all wells was calculated using the following formula:

<u>Fluorescence Ratio</u>=Signal 665 nM/Signal 620 nM

The average ratio of the negative control wells was calculated and subtracted from all wells to account for background signal. Then the fluorescence ratio from each compound treated well was normalized to the fluorescence ratio of the DMSO treated well (100% activity)

ProteinSimple Western Blot Analysis

<u>In vitro</u>

<u>Cell Treatment</u>
Cells were harvested, centrifuged at 1000 rpm for 5 minutes and resuspended in media with 1% FBS. 100 µL of cell suspension was plated into each well of a 96 well plate, corresponding to 30,00 or 40,000 cells per well, depending on the cell line. The plate was incubated overnight at 37 °C with 5% CO_2 to allow the cells to adhere to the plate. The following day, the cells were treated with a dose response, or set dose, of compound using the HP Digital Dispenser for the indicated amount of time. For the 24-hour timepoint, the plate was incubated for 22 hours at 37 °C with 5% CO_2. The media was then removed from the plate and fresh media was added, followed by a second treatment of compound, using the previously described dosing format, and an additional 2-hour incubation at 37 °C with 5% CO_2.

<u>Cell Harvesting and lysate preparation</u>
Following the incubation period, media was removed and cells were lysed with 16 µL of supplemented RIPA buffer. Immediately after harvesting, 8 µL of sample was moved to a PCR strip tube pre-loaded with 2 µL of ProteinSimple 5x Master Mix and the mixture was incubated in the thermocycler for 10 minutes at 95°C. The remaining lysate was used to determine protein

concentration through BCA assay according to manufacturer's instructions (Fisher Scientific, Cat # PI23225). After boiling, using the determined protein concentration, lysates were normalized using 1X sample buffer(5x diluted with prepared RIPA lysis buffer).

In vivo

Tumor samples were collected in bead beater tubes and snap frozen at the time of collection. Samples were kept on ice and each tube was filled 350 µL of lysis buffer(RIPA buffer, Benzonase, $MgSO_4$, HALT and Okadaic Acid), followed by homogenization by bead beating at 4°C for 30 seconds. Following a hard spin to clear insoluble material, 50 µL of sample from each tube was transferred to a 96 well plate and kept on ice. 5 µL of each sample was then transferred to a new well, pre-loaded with 20 µL of lysis buffer and mixed. A BCA assay was then performed on the samples to determine protein concentration. Following normalization with lysis buffer, 8 µL of homogenate was transferred to PCR tubes pre-loaded with 2 µL of 5x sample buffer. The tubes were then briefly mixed and boiled at 95°C for 10 minutes and kept on ice.

Analysis using Jess automated western blotting (in vitro and in vivo)

The lysates were probed with CST #4060 (pAKT(S473)), CST #4370 (pERK1/2(T202/Y204)), CST #4650 (Vinculin)(Loading Control)

In all cases, manufacturer provided Rabbit-HRP secondary antibody, pre-diluted by the manufacturer, was used following incubation with primary antibody.

Each capillary was analyzed on an individual basis by considering the graph of Chemiluminescence relative to expected MW of the proteins being detected. Each protein being detected was then normalized to the Vinculin signal, serving as a loading control, within the capillary using the follow formula:

Normalized Signal: AUC of Protein of Interest (pAKT(473) or pERK1/2 (T202/Y204)/ AUC of Vinculin

Then the normalized signal of each protein of interest from each treated well was normalized to the matching normalized signal from the DMSO treated well (100% activity).

HER2 Immunoprecipitation

Lysates from N87 cells were prepared by washing the cells twice with cold PBS, followed by lysis using M-PER lysis buffer (Thermo Scientific, Cat# 78501) supplemented with Halt protease and phosphatase inhibitor cocktail (Thermo Scientific, Cat# 78441). For immunoprecipitation, 2 mg of protein lysate was incubated with 20 µg of anti-HER2 antibody (CST, Cat# 2165) or anti-IgG control (CST, Cat# 2729) overnight at 4 °C, followed by a 1-hour incubation with protein G magnetic beads (CST, Cat# 70024) on a rotary shaker at room temperature. The antibody–bead conjugates were then pelleted using a magnetic separation rack, washed three times with TBS, and resuspended in sample buffer. The samples were boiled at 95°C for 10 minutes and analyzed using the JESS automated western blot system. The following

antibodies were used: pAKT (S473, CST #4060), pERK1/2 (T202/Y204, CST #4370), p110α (CST #4249), HER2 (CST #2165), HER3 (CST #12708), p85 (CST #4292), and Vinculin (CST #4650).

Traditional Western Blot Analysis

MEFs were pre-treated with DMSO or 500nM VVD-699 for 4 hours, before addition of 100nM 4-hydroxytamoxifen (4HT; Sigma #H7904). Cells were lysed at different time points using 1X Lysis buffer (NEB, #9803S) supplemented with protease (Roche, #4693159001) and phosphatase (Roche, #4906837001) inhibitors.

20 μg of cell lysate was subjected to electrophoresis in 4-12% NuPAGE Bis-Tris gels (Life Technologies) followed by transfer to nitrocellulose membrane. Lysates were probed with phospho-ERK (T202/Y204, #4370), ERK (#9107), phospho-AKT (S473, #9271) and AKT (#2920) from CST and Vinculin (V4505) from Sigma. Bound primary antibodies were incubated with secondary antibodies compatible with infrared detection at 700 nm (# A21058) and 800 nm (# A11369) from Invitrogen. Membranes were scanned using the Odyssey Infrared Imaging System (Odyssey, LICOR).

3D viability assays

Cells were harvested, centrifuged at 1000 rpm for 5 minutes, and resuspended in media with optimized FBS content to promote cell growth. The following conditions were used for the identified cell lines:

10% FBS: FaDu, N87, SK-BR-3, HCC1419, HCC202, KYSE-410, BT-474, SW403, SW948, H358
5% FBS: DLD-1, A549
1% FBS: H460, T84

100 µL of cell suspension was plated into a 96 well low attachment plate and incubated overnight at 37°C to allow the cells to form a sphere. The cells were then treated with a dose-response curve of VVD-699 using the HP Digital Dispenser. The plate was then returned to the 37°C incubator. Every 3 days, the compound was refreshed by treating cells with the same dose response curve of VVD-699, using the HP Digital Dispenser and then returned to the 37°C incubator. For all cell lines except N87, which was harvested on Day 6, 100 µL of 3D CellTiter-Glo (CTG) was added to each well, mixed vigorously for 5 minutes, and then incubated at room temperature for 25 minutes on Day 9. Fluorescence intensity was then measured on the CLARIOstar plate reader (BMG LABTECH, North Carolina, USA).

A549 p110α-Cys^{242}Ser clone generation

Introduction of the Cys^{242}Ser mutation into A549 cells was achieved through CRISPR/Cas9 technology. Briefly, A549 cells were transfected with RNP complexes, containing Cas9 and guide RNA, and ssDNA using the Lonza 4D Nucleofector X unit. After electroporation, cells were moved to a 6 well plate and incubated for 48-72 hours at 37 °C with 5% CO_2. To confirm the Cys^{242}Ser mutation, cells from one well were harvested and subjected to DNA sequencing.

The desired mutation was confirmed at a low frequency, therefore, single cell clones were generated. This was achieved by seeding single cells in a 384 well plate. As the clones grew, they were progressively expanded until enough cells were present to seed a 24 well plate. At this point, cells were again subjected to DNA sequencing, at which point multiple clones carrying homozygous $Cys^{242}Ser$ were identified. Following this confirmation, cells were expanded and banked.

In vitro kinase assay

The assay was performed using Adenosine diphosphate (ADP)-Glo Kinase assay reagents (Promega). It measures kinase activity by quantitating the ADP amount produced from the enzymatic reaction. The luminescent signal from the assay is correlated with the amount of ADP present and is directly correlated with the amount of kinase activity. The compounds were diluted in 2.5 % DMSO and 5 µl of the dilution was added to a 25 µl reaction so that the final concentration of DMSO is 0.5 % in all reactions. The enzymatic reactions were conducted at 30 ^{o} C for 45 minutes. The 25 µl reaction mixture of PI3 kinase contains 40 mM Tris, pH 7.4, 20 mM $MgCl_2$, 0.1 mg/ml BSA, 2.5 µM ATP, kinase substrate and the enzyme. After the enzymatic reaction, 25 µl of ADP-Glo reagent was added and incubated for 45 min at room temperature followed by another 30 min incubation with 50 µl of kinase detection mixture. Luminescence signal was measured using a BioTek *Synergy 2* microplate reader.

Protein expression and purification

The DNA sequence for RBD domain for crystallization (residues 157-300) was synthesized and cloned into pET28b with an N-terminal hexa-histidine SUMO tag. The gene for PI3K residues 105-1048 was synthesized and cloned into pFastBac1 with an N-terminal 6xHis tag followed by a TEV cleavage site. For *E. coli* expression, plasmids were transformed into BL21 DE3 Star. Large scale cultures were grown in 2XYT medium at 37C to an OD600 of 0.6 and induced with 0.4 mM IPTG at 18C overnight. Full lengthPI3KCa was expressed in ExpiSf9 cells using baculovirus mediated expression in the EmBacY viral genome. RBD domain for crystallization was purified using the following procedure. Cells were resuspended in 50 mM HEPES pH 8, 250 mM NaCl, 10% glycerol, 10 mM imidazole and 1mM TCEP and lysed using a Microfluidizer. The lysate was cleared by centrifugation at 25,000 x g for 45 minutes before being loaded onto 5 ml of pre-equilibrated NiNTA resin. The resin was washed with 500 ml lysis buffer and eluted with lysis buffer supplemented with 250 mM imidazole. The SUMO tag was removed with SUMO protease and the sample was dialyzed against lysis buffer. Uncleaved protein was removed with an additional NiNTA purification before additional purification with a Superdex S200 column equilibrated in 25mM HEPES, pH 7.5, 50mM NaCl 3 mM TCEP. Full length p110α was purified in a similar manner, omitting the proteolytic cleavage steps.

Crystallization and structure determination

Purified PI3K RBD was incubated with VVD-442 in 25 mM Tris pH 7.5, 150 mM NaCl, 5% glycerol, 1 mM TCEP, 2% DMSO and the reaction was monitored by intact protein MS. Upon

completion, the protein sample was buffer exchanged into 25 mM Tris pH 7.5, 150 mM NaCl, 1 mM TCEP using a PD-10 desalting column and concentrated to ~12 mg/ml. Crystals were grown in a 1:1 drop of protein to reservoir solution and equilibrated against 4.8 M ammonium acetate, 100 mM MES pH 5.5 at 4C. Crystals were cryoprotected by rapid transfer into reservoir solution supplemented with 25% glycerol before flash freezing in LN2. Diffraction data were collected on Advanced Light Source Beamline 5.0.2 and processed with XDS. The structure was determined by molecular replacement in Phaser using 6VO7 as a search model. The structure was refined using iterative rounds of refinement in REFMAC5 with manual inspection and model building in COOT. Ligand restraints for VVD-442 and the covalent bond with Cys242 were generated in JLigand. Waters were automatically added in COOT and REFMAC5 and manually inspected. Data collection and refinement statistics can be found in Supplementary Table 1.

Intact protein mass spectrometry

To observe covalent ligand engagement of recombinant p110α, samples were analyzed following formic acid quenching on an Agilent LC1290 Infinity II instrument coupled to a 6545 QTOF liquid chromatography–mass spectrometer (Agilent Technologies). A sample volume of 10 µl, equivalent to approximately 1.5 pmol of p110α protein, was injected. The protein was desalted and separated on an AERIS 3.6-µM-wide-bore XB-C8 liquid chromatography column (50×2.1 mm^2, Phenomenex) at 60 °C at a flow rate of 0.5 ml min^{-1}. Liquid chromatography solvent A comprised 0.1% formic acid in 99.9% water, and solvent B was 0.1% formic acid in 99.9% acetonitrile. The column was equilibrated in 10% B for 30 s, followed by a 3.5 min gradient from 10 to 70% B to separate the analytes. This was followed by a 15 s gradient from 70 to 95% B, a 15 s gradient from 95 to 10% B, a 15 s gradient from 10 to 95% B and finally a 15 s gradient from 95 to 10% B to clean the column before re-equilibration. Mass spectra were acquired from 700 to 1,700 Da at a resolution of 25,000. A Dual Agilent Jet Stream Electrospray Ionization Source was used for ionization. The gas temperature was set to 325 °C, with a flow rate of 10 l min^{-1}. The nebulizer was set to 45 pounds per square inch and sheath gas temperature and flow were set to 375 °C and 12 l min^{-1}, respectively. One spectrum was acquired per second with a collision energy of 10 V. The capillary voltage was set to 5,000 V and the nozzle voltage to 2,000 V. The fragmenter, skimmer and octopole radio frequency (RF) peaks were set at 250, 65 and 750 V, respectively. The resulting data files were deconvoluted to protein masses using Agilent MassHunter BioConfirm Software, v.11.0. The biomolecule table containing protein mass and peak intensities was used to quantify the percentage of compound modification relative to the unmodified protein peak, by dividing modified protein intensity by the sum of the unmodified and modified protein intensities. $k_{obs}/[I]$ was calculated using assumptions for pseudo-first-order reaction kinetics (d(VVD-442)/d$t = -k \times$ (VVD-442), (VVD-442)$_t$ = (VVD-442)$_{t0} \times e^{-kt}$) and averaged for each inhibitor concentration; k_{obs} was determined by dividing these values by (I) for each concentration. k_{obs} values were plotted against inhibitor concentrations and the curve was fit with the equation k_{obs} =(k_{inact} *[VVD-442])/(K_i + [VVD-442])

In vivo studies

<u>Human cell-line derived xenograft (CDX) studies</u>: For efficacy studies, following the acclimation period (3-7 days), female immunocompromised mice were inoculated with either

FaDu, A549, H1975, H2122 or H358 cells for tumor development. Details regarding these studies can be found in the table below. Mice were randomized based on tumor volume and were enrolled into different groups. Tumor volumes were measured twice per week after randomization in two dimensions using a caliper. All treatment were administered at the indicated dose level by oral gavage. Characterization of the anti-growth effect of VVD-699 in FaDu xenografts was conducted at Crown Biosciences.

For TE/PD studies, following the acclimation period (3-7 days), immunodeficient NSG or nu/nu mice (Jackson Laboratory) were inoculated subcutaneously into the dorsal flank with a cell suspension of 2 x 10^6 FaDu cells/ mouse in PBS or 1 x 10^6 FaDu cells/ mouse in a 1:1 mixture with Matrigel.

<u>Patient derived xenograft (PDX studies):</u> PDX studies were conducted at Crown Biosciences (BR10564, LU0876, CR2528, CR1554, CR0010 and LU5229) or Champions Oncology (CTG-3196 and CTG-3192). Fresh tumor tissues from mice bearing established primary human cancer PDX model was harvested and cut into small pieces (approximately 2-3 mm in diameter). PDX tumor fragment, harvested from donor mice, was inoculated subcutaneously at the upper right dorsal flank (Crown Biosciences) or left dorsal flank (Champions) into immunocompromised female mice for tumor development. Mice were randomized based on tumor volume (100-200 mm3). After randomization, tumor bearing mice were allocated into indicated treatment groups, with 3 mice per group.

Associated Figure	*Model*	*Injection Site*	*# of Cells/Mouse*	*Cell Suspension*	*Mouse Strain*	*Vendor*	*Age (weeks)*	*Location*
**4A**	FaDu	Right flank	1 x 10^6	0.1 mL (1:1 Geltrex)	Nu/Nu	Jackson Laboratories	6 weeks	Vividion
**4B**	FaDu	Right Flank	2 x 10^6	0.1 mL PBS	NSG	Jackson Laboratories	6 weeks	Vividion
**4C/S4A**	FaDu	Right upper flank	5 x10^6	0.1 mL PBS	NOD/SCID	Zhuhai BesTest Bio-Tech Co.,Ltd	7-9 weeks	Crown Biosciences
**4E**	BR10564	Right upper flank	2-3 mm tumor fragments	N/A	NPG	Vital Star	4-8 weeks	Crown Biosciences
**4G**	LU0876	Right upper flank	2-3 mm tumor fragments	N/A	Nu/Nu	Beijing Anikeeper Biotech Co.,Ltd	6-8 weeks	Crown Biosciences
**4F**	CTG3196	Left flank	Tumor fragments	N/A	Nu/Nu	Charles River	6-8 weeks	Champions Oncology
**4H**	CR2528	Right upper flank	2-3 mm tumor fragments	N/A	Nu/Nu	Beijing Anikeeper Biotech Co.,Ltd	6-8 weeks	Crown Biosciences
**4I**	A549	Right flank	3 x 10^6	0.1 mL (1:1 Geltrex)	NSG	Jackson Laboratories	6 weeks	Vividion
	H1975	Right flank	3 x 10^6	0.1 mL PBS	Nu/Nu	Jackson Laboratories	6-8 weeks	Vividion
**5D**	H2122	Right flank	5 x 10^6	0.1 mL PBS	Nu/Nu	Jackson Laboratories	6-8 weeks	Vividion
**S4C**	CR1554	Right upper flank	2-3 mm tumor fragments	N/A	Nu/Nu	Beijing Anikeeper Biotech Co.,Ltd	6-8 weeks	Crown Biosciences
**S4E**	CR0010	Right upper flank	2-3 mm tumor fragments	N/A	Nu/Nu	Jiangsu GemPharmatech Co.,Ltd	4-8 weeks	Crown Biosciences
	LU5229	Right upper flank	2-3 mm tumor fragments	N/A	Nu/Nu	Jiangsu GemPharmatech Co.,Ltd	4-8 weeks	Crown Biosciences

**S4G**	H358	Right flank	12.5 x 10^6	0.1 mL (1:1Matrigel)	NSG	Jackson Laboratories	6-8 weeks	Vividion
	H2122	Right flank	5 x 10^6	0.1 mL (1:1 Matrigel)	Nu/Nu	Jackson Laboratories	6-8 weeks	Vividion

Geltrex(Cat #12760-021), Matrigel(Cat #354277)

All studies performed at Vividion were performed in compliance with IACUC standards through protocols EB17-010 and EB17-03.

KPAR(^{G12C}): KRAS^{G12C} subcutaneous transplantation experiments were carried out in 8-10 week male and female C57BL/6J mice. 150,000 cells were resuspended in PBS and mixed 1:1 with Geltrex LDEV-Free Reduced Growth Factor matrix (Gibco #1413202) and injected subcutaneously in one flank. For KRAS^{G12C} orthotopic lung tumors 150,000 KPAR^{G12C} cells were resuspended in 100 µl PBS and injected in the tail vein in 8-10 week female C57BL/6J mice. Tumor volume was measured by micro-CT scan as previously described (DOI: 10.1038/s41596-022-00769-5)(*35*).

Glucose timecourse and terminal insulin levels upon multiple doses of Alpelisib or VVD-699: C57BL/6J were dosed orally BID x 2.5 (5 doses total) with vehicle, VVD-699 at 30 or 100 mg/kg and Alpelisib at 19 or 50 mg/kg. Three hours prior last dose, food was withdrawn from the cages to prevent any food-driven glucose production. Basal blood glucose reads were performed 10 minutes before last dose from tail snips using AlphaTRAK 3 glucometer and test strips, and blood glucose levels were then measured 0.5, 1, 2 and 4 hours post last dose from tail snips. At the conclusion of the study, liver and spleen tissues were collected and snap frozen in liquid nitrogen before being stored at -80°C for target engagement analysis through mass spectrometry. To measure plasma insulin levels Terminal blood was collected via cardiac punctures under isoflurane exposure. The resulting plasma was transferred to a clean 96-well plate, sealed, and stored frozen at -80°C until analyses. Insulin levels were determined using commercial mouse insulin ELISA kit (Mercodia), following manufacturer's instructions. In brief, plasma samples were incubated for 2 hours with peroxidase-conjugated anti-insulin antibodies in wells of anti-insulin antibody coated microplate. Unbound enzyme labelled antibodies were removed by a washing step. The bound conjugate was detected by reaction with 3,3',5,5'-tetramethylbenzidine for 15 minutes. The reaction was stopped by adding acid to give a colorimetric endpoint that was read spectrophotometrically at 450 nm on a CLARIOstar plate reader.

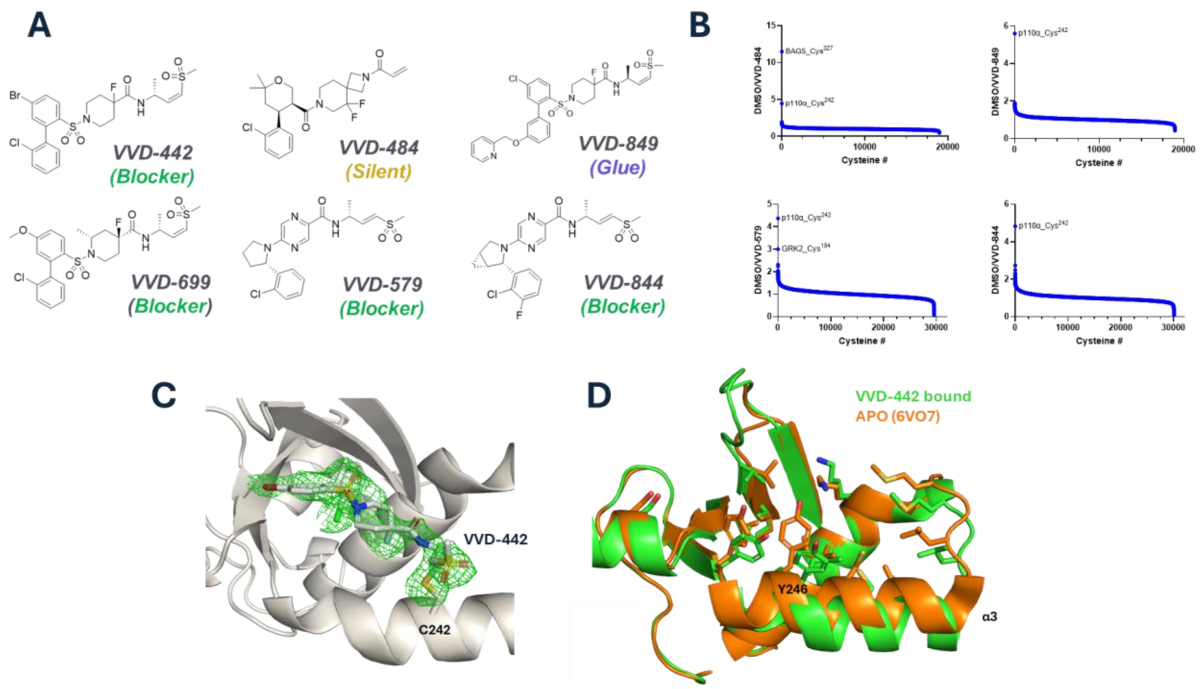

Fig. S1.

(A) Chemical structure of VVD ligands used in the manuscript

(B) Global proteomics selectivity of VVD-484, VVD-849, VVD-579, and VVD-844. Cells were treated with 10 µM of VVD-484, 2 µM of VVD-849 or VVD-579, or 1 µM of VVD-844, which is ~25-fold over TE_{50}s. 20,000-30,000 cysteine containing peptides were measured using TMT quantification for each compound.

(C) F_O-F_C omit map contoured at 2.5 σ showing continuous density for VVD-442 and a covalent bond formed with Cys^{242}.

(D) Superposition of the APO (PDB ID 6VO7) and liganded RAS-binding domain highlighting the shift in α3 and movement of Tyr^{246} required for compound binding.

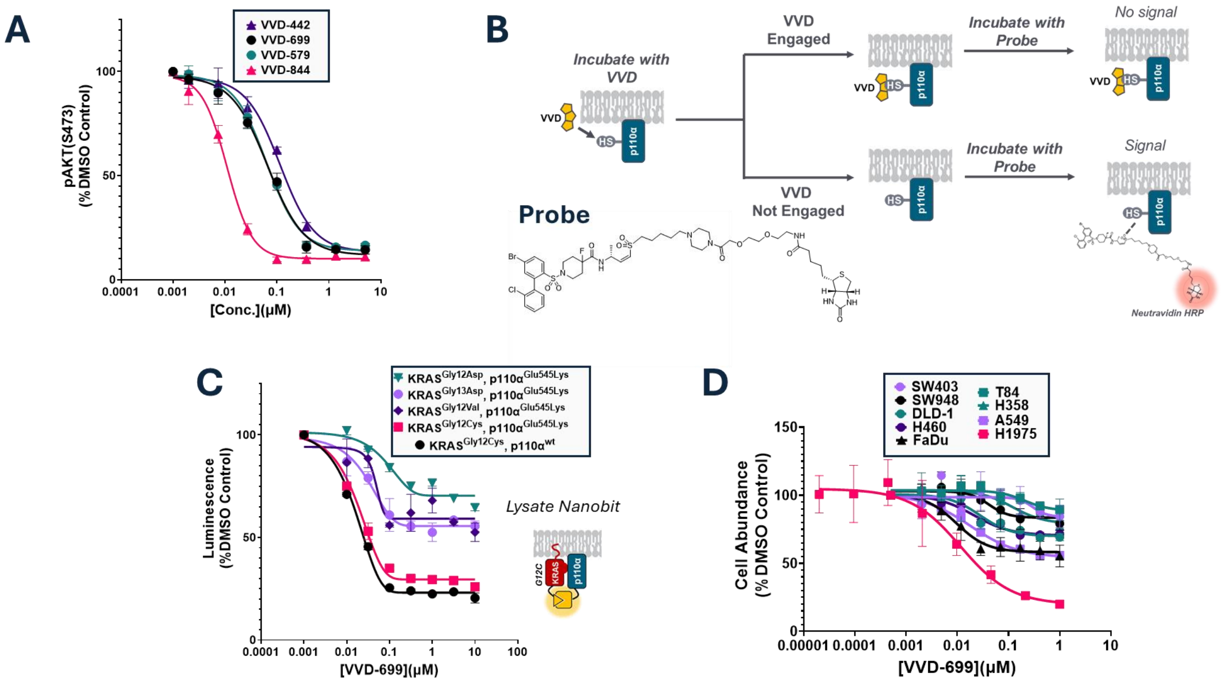

Fig. S2.

(A) H358 cells treated with a dose response of RAS-p110α blockers for 30 minutes, followed by assessment of phosphorylated AKT at Ser^{473} through HTRF(n=3 biological replicates, SD)

(B) Diagram of pocket probe assay used to determine engagement of Cys^{242} on p110α

(C) VVD-699 was screened for its ability to disrupt the interaction between $p110\alpha^{Glu545Lys}$ or $p110\alpha^{WT}$ and different KRAS mutants using the NanoBiT protein-protein interaction assay in HEK293T cell lysates co-transfected with p85α and exposed to compound for 4 hours(n=2 biological replicates, SD).

(D) The ability of VVD-699 to inhibit growth of cells known to have high RAS activation was evaluated through 3D proliferation assays in which cells were treated with VVD-699 every 3 days for 9 days, followed by assessment for cell abundance using Cell Titer Glo(n=2-5 biological replicates per cell line, SD).

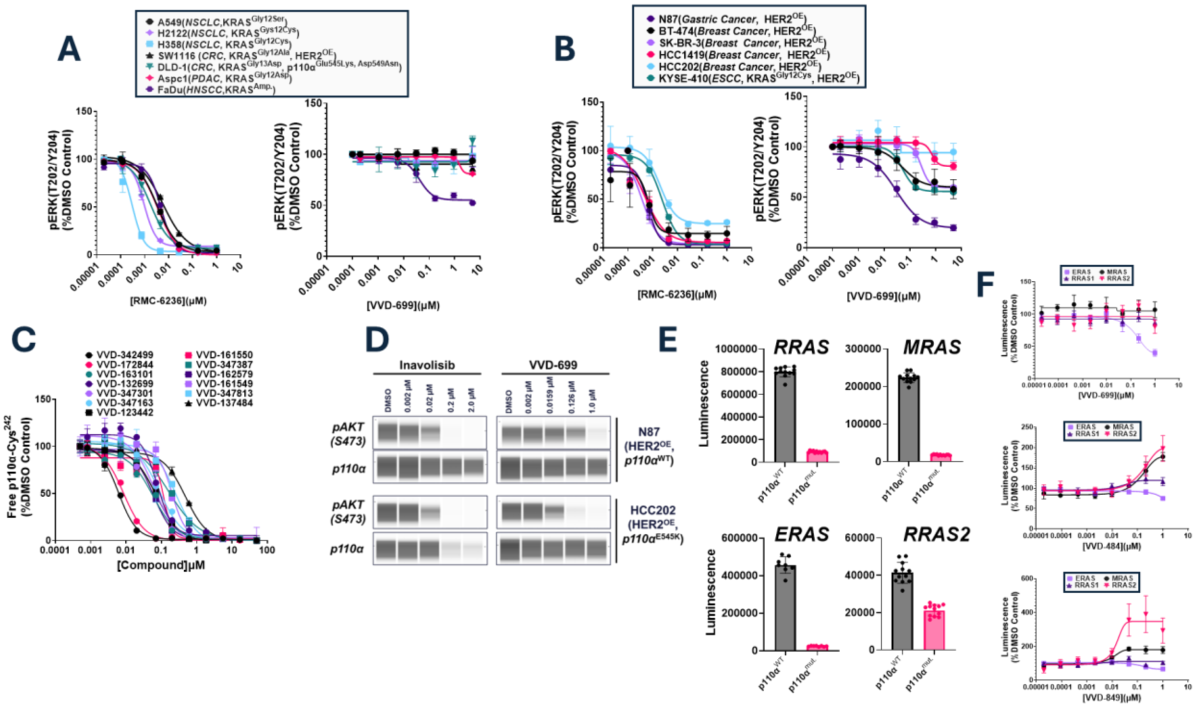

Fig. S3.

(A) KRAS hyperactive cell lines were treated with either RMC-6236 or VVD-699 for 2 hours, followed by assessment of phosphorylated ERK1/2 at Thr^{202}/Tyr^{204}by HTRF(n=2-3 biological replicates per cell line, SD).

(B) HER2 overexpressing cell lines were treated with either RMC-6236 or VVD-699 for 2 hours, followed by assessment of phosphorylated ERK1/2 at Thr^{202}/Tyr^{204} through HTRF(n=3-6 biological replicates per cell line, SD).

(C) Jurkat cell lysates were treated with the indicated compounds for 1 hour, followed by evaluation of binding to p110α-Cy^{s242} by the pocket probe assay(n=2 biological replicates per compound, SD).

(D) N87(HER2^{OE}, p110α^{WT}) or HCC202(HER2^{OE}, p110α^{Glu545Lys}) cells were treated either Inavolisib or VVD-699 for 24 hours. Samples were collected and phosphorylated AKT at Ser^{473}and p110α levels were determined through Protein Simple western blotting.

(E/F) The interaction between the identified RAS isoforms and p110α^{WT} or p110α$^{mut.}$ using the NanoBiT protein-protein interaction assay in H358 cells in which luminescence reflects interaction between the proteins (E)(n=8-12 biological repeats per RAS related isoform, SD). Using this same assay format, the ability of VVD-849, VVD-484 or VVD-699 to impact the interaction between the identified RAS related isoforms and p110α^{WT} was determined (F)(n=2-8 biological repeats per RAS related isoform, SD).

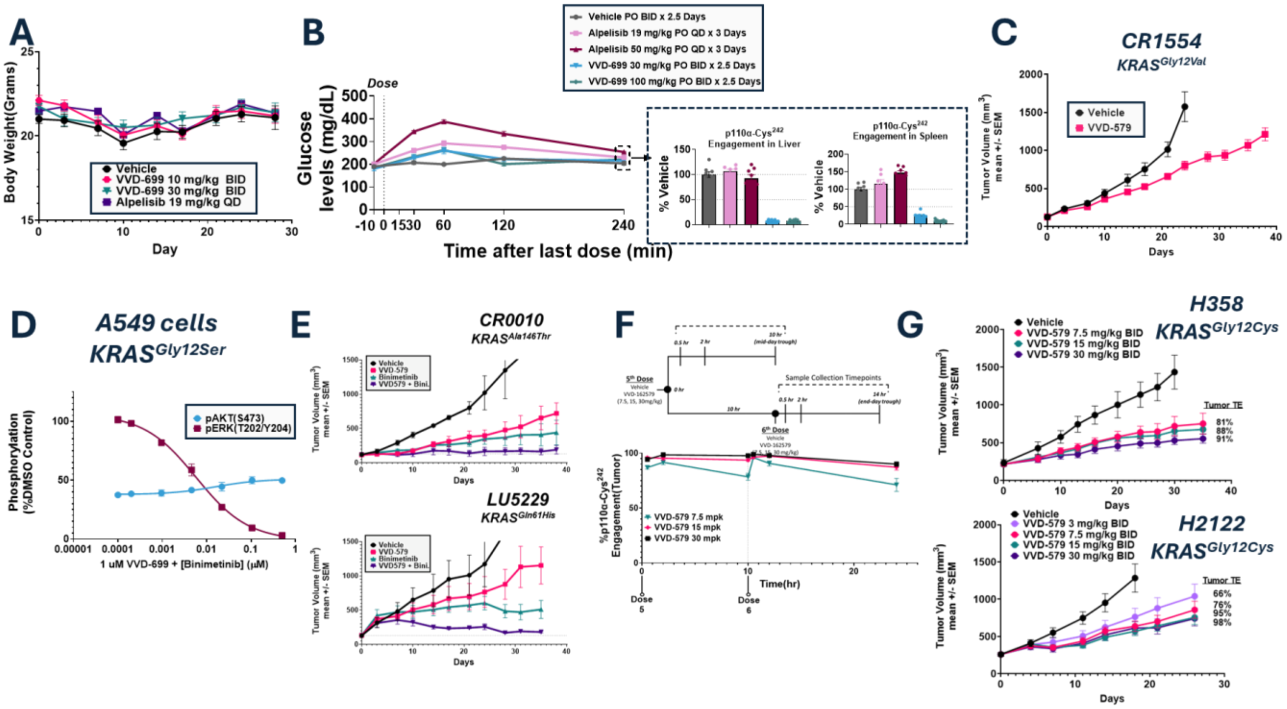

Fig. S4.

(A) Body weights of mice bearing FaDu(KRAS^{amp}) xenografts that were treated with the indicated doses of VVD-699 or alpelisib. Data are shown as mean ± SEM; n=10 animals/group for vehicle and alpelisib treated animals, n=9 animals/group for 10 & 30 mg/kg VVD-699 treated animals.

(B) Mice were administered the indicated doses of alpelisib or VVD-699 for 3 days, followed by serial measurement of blood glucose levels for 4 hours post-last dose. At the conclusion of the time-course, spleen and liver were collected from the animals and compound binding to Cys^{242} on p110α was determined by mass spectrometry.

(C) Anti-tumor efficacy of VVD-579 in KRAS^{Gly12Val} PDX model CR1554. Data are shown as mean ± SEM; n=3 animals/group. Mice were dosed orally BID with 30 mg/kg VVD-579.

(D) A549(KRAS^{Gly12Ser}) cells treated with a fixed 1 μM dose of VVD-699 and a dose response of binimetinib for 2 hours. Cells were harvested and activation of the PI3K/AKT pathway (using phosphorylated AKT at Ser^{473}) and MAPK pathway(using phosphorylated ERK1/2 at Thr^{202}/Tyr^{204}) pathways were assessed by western blot(n=2 biological replicates, SD).

(E) Anti-tumor efficacy of VVD-579, binimetinib, or a combination of both in CR0010(KRAS^{Ala146Thr}) (Top) or LU5229(KRAS^{Gln61His}) (Bottom) PDX models. Data are shown as mean ± SEM; n=3 animals/group. Mice were dosed orally BID with 30 mg/kg VVD-579, QD with 30 mg/kg binimetinib or a combination of both.

(F) Mice bearing H358 xenografts were orally administered the indicated dose levels of VVD-579 with a BID dosing schedule. After receiving either 5 or 6 doses of VVD-579, tumor samples were collected at the indicated timepoints and compound binding to Cys^{242} on p110α was determined by mass spectrometry. Data are shown as mean ± SEM; n=5 animals/group, except the 2- and 10.5-hour groups dosed at 30 mg/kg VVD-579 in which n=4 animals/group.

(G) Anti-tumor efficacy of VVD-579 in H358($KRAS^{Gly12Cys}$) or H2122($KRAS^{Gly12Cys}$) xenografts. Data are shown as mean ± SEM; n=10 animals/group. Mice were dosed orally BID with the indicated dose levels of VVD-579.

BID, twice daily; QD, once daily; SEM, standard error of the mean.

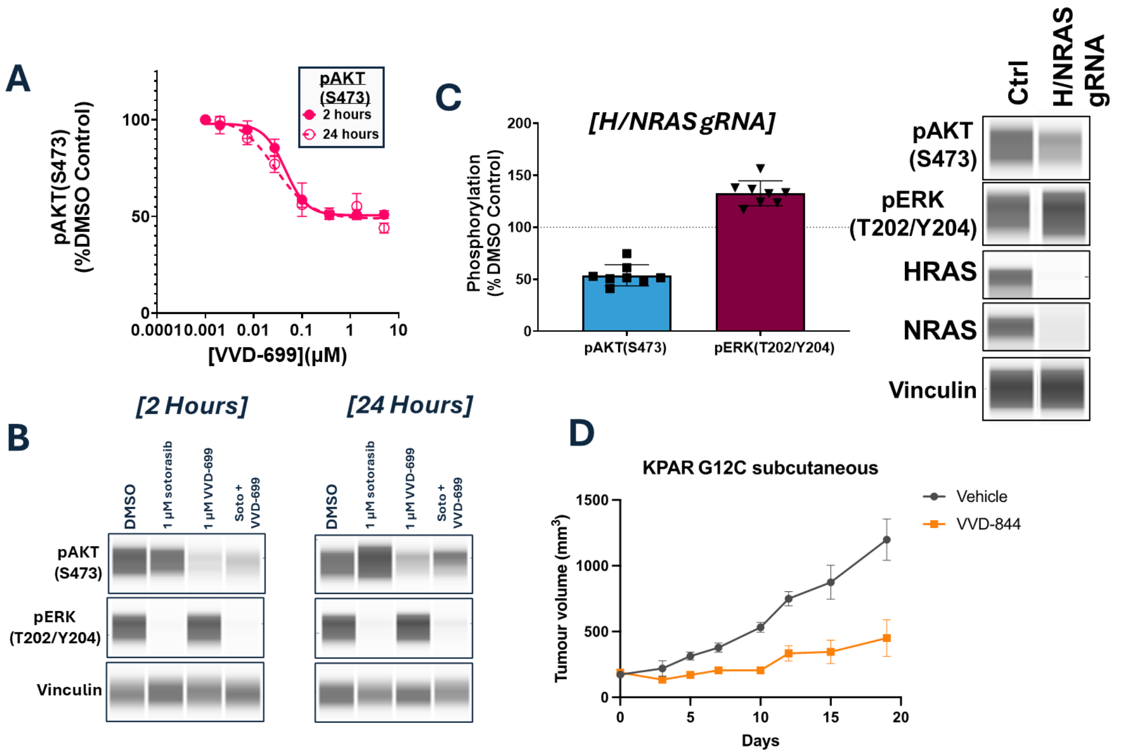

Fig. S5.

(A) H2122(KRAS^{Gly12Cys}) cells were treated with a dose response of VVD-699 for 2 or 24 hours. At each timepoint, samples were collected and phosphorylated AKT at Ser^{473} was measured by HTRF(n=3 biological repeats, SD).

(B) H2122(KRAS^{Gly12Cys}) cells were treated with indicated compounds for 2 or 24 hours. Samples were collected and activation of the PI3K/AKT (using phosphorylated AKT at Ser^{473}) and MAPK (using phosphorylated ERK1/2 at Thr^{202}/Tyr^{204}) pathways were assessed by Protein Simple western blot.

(C) H2122(KRAS^{Gly12Cys}) cells were transfected with CRISPR control guides or guides targeting HRAS and NRAS for 72-96 hours. Samples were collected and activation of the PI3K/AKT (using phosphorylated AKT at Ser^{473}) and MAPK (using phosphorylated ERK1/2 at Thr^{202}/Tyr^{204}) pathways were assessed by western blot. Knockdown of HRAS and NRAS was also confirmed by Protein Simple western blotting(n=8 biological repeats, SD).

(D) Anti-tumor efficacy of VVD-844 in subcutaneous KPAR^{Gly12Cys} tumors. Data are shown as mean ± SEM; n=8-9 mice/group. Mice were dosed orally QD with 10 mg/kg VVD-844.

QD, once daily; SEM, standard error of the mean.

	k_{inact}	K_i	k_{inact}/K_i	Mass Spec.	Pocket Probe	$KRAS^{G12C}$:p110α Lysate Nanobit		pAKT(S473) [H358 cells]	
ID	s^{-1}	μM	$M^{-1} s^{-1}$	TE_{50} (μM)	TE_{50} (μM)	IC_{50} (μM)	I_{max} (%DMSO)	IC_{50} (μM)	I_{max} (%DMSO)
VVD-442	ND	ND	ND	0.089	0.11	0.11[a]	76%[a]	0.115	85%
VVD-699	0.018	40.7	445	0.05	0.075	0.036[a]	78%[a]	0.066	86%
VVD-579	ND	ND	ND	0.013	0.06	0.03[a]	75%[a]	0.063	84%
VVD-844	ND	ND	ND	0.003	0.009	0.004[a]	86%[a]	0.011	90%
VVD-484	ND	ND	ND	0.44	0. 50	0.59[b]	-77%[b]	ND	ND
VVD-849	ND	ND	ND	0.063	0.1	0.02[a]	-378%[a]	0.04	-379%

[a] 4 hour compound incubation, p85α co-transfected
[b] 1 hour compound incubation

Table S1

Summary of VVD ligands described in this manuscript. K_i and k_{inact} values for VVD-699 determined with recombinant p110α using intact protein MS. All additional values determined using methodology described elsewhere in this manuscript. H358 cells were treated with compound for 30 minutes prior to collection and evaluation of phosphorylated AKT at Ser^{473} was measured by HTRF.

Table S2

PI3K-α RAS binding domain in complex with VVD-442<br>PDB ID 9E8M	
**Data collection**	
**Wavelength (Å)**	**1.00003**
**Resolution (Å)**	**47.8- 2.83 (2.93 -2.83)[1]**
**Space group**	**$C222_1$**
**Unit cell (Å)**	**62.4 88.3 139.0**
**Unique reflections**	**9477 (916)**
**Multiplicity**	**6.6 (6.9)**
**Completeness**	**99.9 (100)**
**Mean I/sigma(I)**	**7.4 (1.7)**
**Wilson B-factor**	**41.3**
**Rsym**	**0.237 (1.14)**
**Rpim**	**0.099 (0.47)**
**CC1/2**	**0.986 (0.608)**
**Refinement**	
**Reflections using in refinement**	**9471 (916)**
**Reflections used for Rfree**	**485 (46)**
**R-work**	**0.191**
**R-free**	**0.268**
**RMS (bonds, Å**	**0.009**
**RMS (angles, °)**	**1.74**
**Ramachandran allowed (%)**	**98.5**
**Ramachandran outliers (%)**	**1.5**
**Average B-factor**	**41**
**Protein**	**42**
**Ligand**	**56**
**Water**	**30**

1. Values in parenthesis include only reflections from the highest resolution shell.

References:

1. S. R. Punekar, V. Velcheti, B. G. Neel, K. K. Wong, The current state of the art and future trends in RAS-targeted cancer therapies. *Nat Rev Clin Oncol* **19**, 637-655 (2022).
2. P. Rodriguez-Viciana *et al.*, Phosphatidylinositol-3-OH kinase as a direct target of Ras. *Nature* **370**, 527-532 (1994).
3. P. H. Warne, P. R. Viciana, J. Downward, Direct interaction of Ras and the amino-terminal region of Raf-1 in vitro. *Nature* **364**, 352-355 (1993).
4. J. A. Engelman *et al.*, Effective use of PI3K and MEK inhibitors to treat mutant Kras G12D and PIK3CA H1047R murine lung cancers. *Nat Med* **14**, 1351-1356 (2008).
5. M. Zhang, H. Jang, R. Nussinov, The structural basis for Ras activation of PI3Kα lipid kinase. *Physical Chemistry Chemical Physics* **21**, 12021-12028 (2019).
6. E. Castellano *et al.*, Requirement for Interaction of PI3-Kinase p110α with RAS in Lung Tumor Maintenance. *Cancer Cell* **24**, 617-630 (2013).
7. S. Gupta *et al.*, Binding of Ras to Phosphoinositide 3-Kinase p110α Is Required for Ras-Driven Tumorigenesis in Mice. *Cell* **129**, 957-968 (2007).
8. S. E. Nunnery, I. A. Mayer, Management of toxicity to isoform α-specific PI3K inhibitors. *Ann Oncol* **30**, x21-x26 (2019).
9. B. Vanhaesebroeck, M. W. D. Perry, J. R. Brown, F. André, K. Okkenhaug, PI3K inhibitors are finally coming of age. *Nat Rev Drug Discov* **20**, 741-769 (2021).
10. F. André *et al.*, Alpelisib for PIK3CA-Mutated, Hormone Receptor-Positive Advanced Breast Cancer. *N Engl J Med* **380**, 1929-1940 (2019).
11. D. Czyzyk *et al.*, Structural insights into isoform-specific RAS-PI3Kα interactions and the role of RAS in PI3Kα activation. *Nat Commun* **16**, 525 (2025).
12. M. Zhang, H. Jang, R. Nussinov, PI3K Driver Mutations: A Biophysical Membrane-Centric Perspective. *Cancer Res* **81**, 237-247 (2021).
13. K. A. Baltgalvis *et al.*, Chemoproteomic discovery of a covalent allosteric inhibitor of WRN helicase. *Nature* **629**, 435-442 (2024).
14. R. Cooley *et al.*, Development of a cell-free split-luciferase biochemical assay as a tool for screening for inhibitors of challenging protein-protein interaction targets. *Wellcome Open Res* **5**, 20 (2020).
15. N. G. Martinez *et al.*, Biophysical and Structural Characterization of Novel RAS-Binding Domains (RBDs) of PI3Kα and PI3Kγ. *J Mol Biol* **433**, 166838 (2021).
16. M. Molina-Arcas *et al.*, Development of combination therapies to maximize the impact of KRAS-G12C inhibitors in lung cancer. *Sci Transl Med* **11**, (2019).
17. M. Dajee, M. Tarutani, H. Deng, T. Cai, P. A. Khavari, Epidermal Ras blockade demonstrates spatially localized Ras promotion of proliferation and inhibition of differentiation. *Oncogene* **21**, 1527-1538 (2002).
18. A. Majumder *et al.*, The role of HER2 and HER3 in HER2-amplified cancers beyond breast cancers. *Sci Rep* **11**, 9091 (2021).

19. M. M. Murillo *et al.*, Disruption of the Interaction of RAS with PI 3-Kinase Induces Regression of EGFR-Mutant-Driven Lung Cancer. *Cell Rep* **25**, 3545-3553.e3542 (2018).
20. S. R. Rangan, A new human cell line (FaDu) from a hypopharyngeal carcinoma. *Cancer* **29**, 117-121 (1972).
21. J. Jiang *et al.*, Translational and Therapeutic Evaluation of RAS-GTP Inhibition by RMC-6236 in RAS-Driven Cancers. *Cancer Discov* **14**, 994-1017 (2024).
22. K. W. Song *et al.*, RTK-Dependent Inducible Degradation of Mutant PI3Kα Drives GDC-0077 (Inavolisib) Efficacy. *Cancer Discov* **12**, 204-219 (2022).
23. M. H. Hofmann, D. Gerlach, S. Misale, M. Petronczki, N. Kraut, Expanding the Reach of Precision Oncology by Drugging All KRAS Mutants. *Cancer Discov* **12**, 924-937 (2022).
24. P. A. Jänne *et al.*, Selumetinib Plus Docetaxel Compared With Docetaxel Alone and Progression-Free Survival in Patients With KRAS-Mutant Advanced Non-Small Cell Lung Cancer: The SELECT-1 Randomized Clinical Trial. *Jama* **317**, 1844-1853 (2017).
25. A. J. de Langen *et al.*, Sotorasib versus docetaxel for previously treated non-small-cell lung cancer with KRAS(G12C) mutation: a randomised, open-label, phase 3 trial. *Lancet* **401**, 733-746 (2023).
26. M. M. Awad *et al.*, Acquired Resistance to KRAS(G12C) Inhibition in Cancer. *N Engl J Med* **384**, 2382-2393 (2021).
27. J. Canon *et al.*, The clinical KRAS(G12C) inhibitor AMG 510 drives anti-tumour immunity. *Nature* **575**, 217-223 (2019).
28. M. B. Ryan *et al.*, KRAS(G12C)-independent feedback activation of wild-type RAS constrains KRAS(G12C) inhibitor efficacy. *Cell Rep* **39**, 110993 (2022).
29. J. Boumelha *et al.*, An Immunogenic Model of KRAS-Mutant Lung Cancer Enables Evaluation of Targeted Therapy and Immunotherapy Combinations. *Cancer Res* **82**, 3435-3448 (2022).
30. S. Suire *et al.*, Gbetagammas and the Ras binding domain of p110gamma are both important regulators of PI(3)Kgamma signalling in neutrophils. *Nat Cell Biol* **8**, 1303-1309 (2006).
31. P. Rodriguez-Viciana, P. H. Warne, B. Vanhaesebroeck, M. D. Waterfield, J. Downward, Activation of phosphoinositide 3-kinase by interaction with Ras and by point mutation. *Embo j* **15**, 2442-2451 (1996).
32. G. Q. Gong *et al.*, A small-molecule PI3Kα activator for cardioprotection and neuroregeneration. *Nature* **618**, 159-168 (2023).
33. D. Prakoso *et al.*, Gene therapy targeting cardiac phosphoinositide 3-kinase (p110α) attenuates cardiac remodeling in type 2 diabetes. *Am J Physiol Heart Circ Physiol* **318**, H840-h852 (2020).
34. M. E. Kavanagh *et al.*, Selective inhibitors of JAK1 targeting an isoform-restricted allosteric cysteine. *Nat Chem Biol* **18**, 1388-1398 (2022).
35. M. Zaw Thin *et al.*, Micro-CT acquisition and image processing to track and characterize pulmonary nodules in mice. *Nat Protoc* **18**, 990-1015 (2023).
